# The Human Nasal Microbiota and *Staphylococcus aureus* Carriage

**DOI:** 10.1371/journal.pone.0010598

**Published:** 2010-05-17

**Authors:** Daniel N. Frank, Leah M. Feazel, Mary T. Bessesen, Connie S. Price, Edward N. Janoff, Norman R. Pace

**Affiliations:** 1 Department of Molecular, Cellular, and Developmental Biology, University of Colorado at Boulder, Boulder, Colorado, United States of America; 2 Mucosal and Vaccine Research Program Colorado, Aurora, Colorado, United States of America; 3 Department of Veterans Affairs Medical Center, Denver, Colorado, United States of America; 4 University of Colorado School of Medicine, Aurora, Colorado, United States of America; 5 Division of Infectious Diseases, Denver Health Medical Center, Denver, Colorado, United States of America; Cairo University, Egypt

## Abstract

**Background:**

Colonization of humans with *Staphylococcus aureus* is a critical prerequisite of subsequent clinical infection of the skin, blood, lung, heart and other deep tissues. *S. aureus* persistently or intermittently colonizes the nares of ∼50% of healthy adults, whereas ∼50% of the general population is rarely or never colonized by this pathogen. Because microbial consortia within the nasal cavity may be an important determinant of *S. aureus* colonization we determined the composition and dynamics of the nasal microbiota and correlated specific microorganisms with *S. aureus* colonization.

**Methodology/Principal Findings:**

Nasal specimens were collected longitudinally from five healthy adults and a cross-section of hospitalized patients (26 *S. aureus* carriers and 16 non-carriers). Culture-independent analysis of 16S rRNA sequences revealed that the nasal microbiota of healthy subjects consists primarily of members of the phylum Actinobacteria (e.g., *Propionibacterium spp.* and *Corynebacterium spp.*), with proportionally less representation of other phyla, including Firmicutes (e.g., *Staphylococcus spp.*) and Proteobacteria (e.g. *Enterobacter spp*). In contrast, inpatient nasal microbiotas were enriched in *S. aureus* or *Staphylococcus epidermidis* and diminished in several actinobacterial groups, most notably *Propionibacterium acnes*. Moreover, within the inpatient population *S. aureus* colonization was negatively correlated with the abundances of several microbial groups, including *S. epidermidis* (p = 0.004).

**Conclusions/Significance:**

The nares environment is colonized by a temporally stable microbiota that is distinct from other regions of the integument. Negative association between *S. aureus*, *S. epidermidis*, and other groups suggests microbial competition during colonization of the nares, a finding that could be exploited to limit *S. aureus* colonization.

## Introduction

*Staphylococcus aureus* is an invasive human pathogen with increasing incidence and morbidity in hospitals and the community. Both healthy persons and those with underlying illness are at risk for diverse skin and soft tissue infections, endocarditis, osteomyelitis, meningitis, bacteremia, and pneumonia (including pneumonia arising as a complication of influenza [1]), with mortality rates ranging from 6–40% [2], [3]. The high frequency of poorly responsive and recurrent *S. aureus* disease in apparently immunocompetent hosts is a challenging feature of these infections [4]. Groups that are particularly susceptible include children in daycare [5], sports teams [6], [7], [8], [9], jailed inmates [10], [11], [12], and military personnel [13], [14], [15], [16]. Moreover, the emergence and rapid spread of methicillin-resistant *S. aureus* (MRSA) has placed substantial burden on the healthcare system.

Colonization of the nares is a potent and increasingly prevalent risk factor for subsequent *S. aureus* infection [17], [18], [19], [20]. In at least 80% of *S. aureus* bacteremia cases in colonized subjects, the infecting strain is identical to a nasal colonizing strain detected prior to onset of bacteremia [17], [21]. Followed longitudinally, approximately 20–30% of persons are colonized persistently with *S. aureus*, 30% are colonized intermittently, and 50% never, or rarely, are colonized [22], [23]. Why some individuals apparently are resistant to colonization, and thus at lower risk of infection, remains an open question. Understanding the biology of this pathogen, especially its ecological niche in humans and the initial step in infection, colonization, may therefore provide new modalities to limit pathogenesis.

*S. aureus* carriage is influenced by myriad host and environmental factors [24], [25]. To establish itself in the nares, *S. aureus* must successfully compete with many co-occurring microorganisms, including corynebacteria, coagulase-negative staphylococci, and *Streptococcus pneumoniae* [26]. We hypothesize that competition and cooperation between *S. aureus* and nares-associated microbial communities directly impacts the incidence and prevalence of *S. aureus* colonization and subsequent infection. Although prior studies have analyzed associations between *S. aureus* and other well-characterized microorganisms [27], [28], [29], the microbial consortia that normally inhabit the nasal cavity may be more complex than indicated by traditional microbiological culture. Consequently, the microbial ecology of *S. aureus* colonization likely is incompletely understood. To surmount these potential limitations, we used culture-independent analyses of 16S ribosomal RNA sequences to more fully characterize the repertoire of indigenous microbial communities within the human nares of healthy and hospitalized adults in relation to *S. aureus* colonization.

## Results

### Study Design

**Figure 1:**
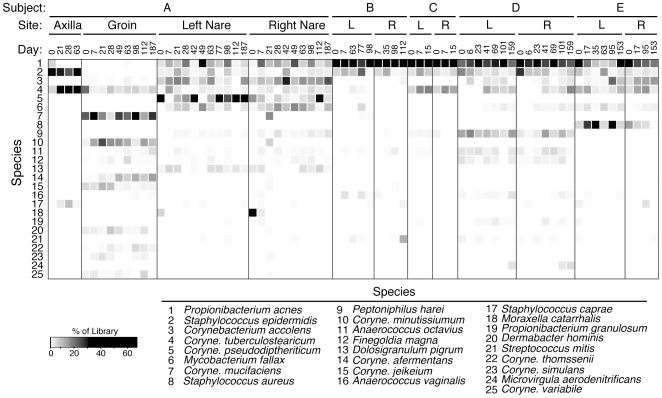
Relative abundance of predominant bacterial taxa followed longitudinally in healthy adults. Shading indicates the proportion of each rDNA library represented by a particular rDNA sequence type. Rows represent species or genus-level taxonomic groups and columns represent individual specimens. Data are presented only for the 25 most abundant taxa, which account for 90% of rDNA sequences analyzed.

To determine the frequency, diversity, and temporal stability of resident microbial communities, we collected nasal specimens longitudinally from the left and right nares of five healthy adults (Subjects A–E) over the course of 2–24 weeks (timepoints are listed in Fig. 1). For comparison, axilla, groin, and nasal specimens were collected in parallel from one individual (Subject A) to assess whether the nares harbor the same types of microorganisms as other regions of the integument. All healthy adults showered daily, 4/5 used antiperspirants and 1/5 used deodorant daily.

Hospitalized adults were sampled in a case-control study of 20 MRSA-colonized patients and 24 patients without MRSA colonization (based on routine nasal swab culture) while admitted to the intensive care units in two hospitals. Colonization was identified by results of culture of both nares with nasal swabs inoculated onto CHROMagar (Materials and Methods). Because patients without MRSA by culture could be either colonized with methicillin-sensitive *S. aureus* (MSSA) or not colonized by *S. aureus,* DNA prepared from nasal swabs was subjected to *S. aureus*-specific *femA* gene PCR [30] to detect all *S. aureus* (i.e., MSSA and MRSA). Two specimens were excluded on the basis of poor DNA recovery (i.e., negative rDNA PCR results, described below). All MRSA culture-positive patients were positive for *S. aureus* by *femA* PCR, indicating a low false negative rate (<1/16) of this PCR assay. Patients with negative cultures for MRSA and positive *S. aureus* PCR results were presumptively classified as MSSA. In aggregate, 18 inpatients were classified as MRSA carriers, 8 as MSSA carriers, and 16 *S. aureus* non-colonized.

### Culture-Independent Microbe Identification

Bacteria present in specimens were identified by phylogenetic analysis of rDNA sequences, amplified from DNA isolated from swabs by PCR with pan-bacterial primers. Among healthy adults, PCR amplification was successful in 63/74 (85%) nasal specimens and 9/9 (100%) groin specimens, but only 4/11 (36%) axilla specimens. The failure of some samples to amplify was likely due to insufficient biomass. Although sampling methods could have been optimized to increase biomass (e.g., through wet-swabs or lavage), we followed the standard procedures used in the hospitals in order to compare data from all specimens. PCR successfully detected bacterial 16S rDNA in 26/28 (93%) inpatients colonized with *S. aureus* and 16/16 (100%) without *S. aureus* colonization.

Broad-range rDNA amplicons were subjected to high-throughput pyrosequencing on the Roche 454 GS-FLX platform. The reverse rRNA PCR primer (SSU338R [31]) included a barcode sequence unique to each specimen in order to amplify and quantify multiple bacterial 16S rDNA sequences simultaneously (multiplex pyrosequencing reactions) [32]. To maximize the accuracy of bacterial identification, we rigorously screened sequences to remove poor quality reads. The resulting dataset consisted of 33,289 polished sequences, with a median length of 237 nucleotides (range 177–268 nt.) and a mean of 268 sequences per specimen. Good's coverage values ranged from 88%–100% per subject (mean 98%) therefore the true biodiversity of each specimen was adequately sampled.

rRNA sequences were provisionally identified by BLAST query of 16S rRNA sequences extracted from the All-Species Living Tree Project database (version LTP_S95; [33]) and results corroborated through the RDP Classifier tool [34], [35], parsimony insertion into the SILVA SSURef_98 tree [36], and pairwise distance comparisons to aligned staphylococcal sequences. Although the LTP database contains only a fraction of the 16S rRNA sequences present in GenBank or EMBL, its focus on type strains, rather than environmental sequences, can provide more accurate taxonomic assignments of human-associated bacterial rDNA, which typically are closely related to well-characterized microbial taxa [37], [38], [39]. Indeed, 91% of the rRNA sequences were assigned to the species-level (BLAST %ID scores ≥97%) and 93% assigned to the genus-level (BLAST %ID scores ≥95%). The remaining sequences were classified to higher taxonomic levels. Excellent concordance was observed between BLAST, RDP, and SILVA classifications (<0.1% of sequences had discordant results, which were resolved in favor of matches between two of the three methods).

### Ecology of the Healthy Nares Microbiota

The majority of nares rRNA sequences obtained from healthy individuals belonged to only two bacterial phyla, the Actinobacteria (i.e., High-G+C Gram positive organisms such as corynebacteria; 68% of sequences) and Firmicutes (i.e., Low-G+C Gram positive organisms such as staphylococci; 27% of sequences; Table 1 and Table S1). Proteobacteria (4.%), Bacteroidetes (1.4%), Fusobacteria (0.21%), Cyanobacteria (0.08%), Tenericutes (0.07%) and Deinococcus (0.01%) accounted for the remainder of phylum-level diversity. Members of the Actinobacteria and Firmicutes also dominated specimens obtained from the groin and axilla (Fig. 1, Table 1, Table S1). Similar distributions of predominant phyla have been reported for integument-associated microbiotas of healthy individuals [37], [40], [41] and those with chronic wounds [39].

**Table 1:** Nares-associated bacterial diversity in healthy and hospitalized adults^1^.

		Inpatient		
		*S. aureus* Carriage^2^		
Top Blast Hit^3^	Neg	Pos	*p* 4	Healthy
**Firmicutes**	**54.0%** 5	**66.5%** 5		**25.6%** 5
* Staphylococcus aureus*	*0.1*	*46.1*	*< 0.001*	*4.5*
* Staphylococcus epidermidis*	*43.7*	*8.2*	*0.004*	*10.3*
* Peptoniphilus spp.*	*1.8*	*1.3*		*3.2*
* Anaerococcus spp.*	*1.1*	*1.0*		*1.4*
Other Firmicutes	*7.3*	*9.8*		*6.2*
**Actinobacteria**	**34.4**	**14.9**	**0.06**	**69.0**
* Propionibacterium acnes*	*12.2*	*3.4*		*42.4*
* Corynebacterium accolens*	*5.6*	*5.2*		*7.3*
* Corynebacterium pseudodiphtheriticum*	*0.0*	*1.9*		*4.8*
* Corynebacterium tuberculostearicum*	*5.7*	*1.0*		*8.0*
* Mycobacterium spp.*	*0.1*	*0.2*		*3.4*
Other Actinobacteria	*10.8*	*3.1*		*3.1*
**Proteobacteria**	**8.6**	**17.0**		**4.0**
* Enterobacter ludwigii*	*0.1*	*5.3*		*0.1*
Other Proteobacteria	*8.5*	*11.7*		*3.9*
**Bacteroidetes**	**1.11**	**0.34**		**1.36**
**Fusobacteria**	**0.08**	**0.02**		**0.21**
**Cyanobacteria**	**0.02**	**0.02**		**0.08**
**Deinococcus**	**0.02**	**0.00**		**0.01**
**Tenericutes**	**0.04**	**0.00**		**0.07**
** Sequences** 6 **:**	5227	9095		16411
** Subjects** 7 **:**	16	26		5
** Specimens** 8 **:**	16	26		63
** Observed 99% OTUs** 9 **:**	36.8	34.7		52.4
** Estimated 99% OTU richness** 10 **:**	108.0	113.8		244.6
** Shannon diversity:**	3.5	3.4		4.5
** Shannon evenness:**	68%	67%		79%
** Simpson diversity:**	6.6	6.9		14.1

1 Table summarizes most abundant species/genera 0f 16S rRNA sequences in anterior nares swabs. See supplemental information for complete dataset.

2 *S. aureus* non-colonized (Neg) with colonized (Pos) ICU patients, classified by culture and *fem*A gene PCR.

3 Inferred from highest bit-score in BLAST query. Blast %IDs <97 are named only to the genus level. Sequence abundances are listed for phyla (bold) and the 10 most abundant species/genera (italics; 90% of total sequences).

4 p-value for Student's t-test comparison of colonized and non-colonized patients. Only values <0.1 are noted.

5 Abundance of sequences for each category. Values are averages for subjects in a category.

6 Number of sequences analyzed in each category.

7 Subjects included in category.

8 Specimens included in category. If more than one specimen/subject were analyzed, mean rRNA abundances were weighted by the number of specimens so that all subjects contribute equally to the mean value.

9 Operational taxonomic units (OTUs) were assembled by complete-linkage clustering at a threshold of 99% uncorrected sequence identity. Tabulated values are means obtained through 1000 boot-strap replicates using rarefaction to normalize for differences in sampling efficiency.

10 Schao1 non-parametric estimate of species richness for 99% OTUs.

**Figure 2:**
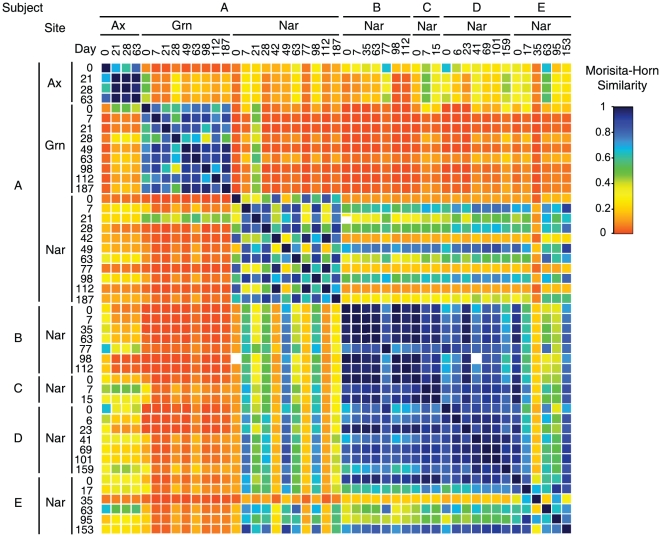
Similarities between microbiotas determined longitudinally for healthy adults. Morisita-Horn Community Similarity Indices (C_MH_) were calculated for each pairwise combination of samples and plotted as a heatmap that compares all values. Color gradient denotes C_MH_ values, which range from 0.0 (no similarity between communities) to 1.0 (identical communities). Ax: Axilla samples. Grn: Groin samples. Nar: Nares samples.

Although the precise distributions of species-level microbial groups differed from specimen to specimen, consistent patterns of microbial taxa were observed over time within each individual and anatomical location (Fig. 1, Fig. 2). For instance, all of the longitudinal groin specimens from Subject A were dominated by corynebacterial species (e.g. *C. mucifaciens, C. minutissiumum*) that were not present, or much less abundant, in the axilla and nares samples of Subject A, including specimens collected on the same day. Similarly, sequences representative of *Propionibacterium acnes* were prevalent in the longitudinal nares samples of all subjects, but were observed far less frequently in axilla and groin swabs. *Staphylococcus epidermidis* was prevalent in the nares and axilla specimens, but not the groin, whereas *S. aureus* was observed only in the nares of two individuals. Specimens collected at the same time point from the left and right nares of the same individual did not differ appreciably in community composition relative to one another (Fig. 1), therefore these sequences were pooled for subsequent analyses.

**Figure 3:**
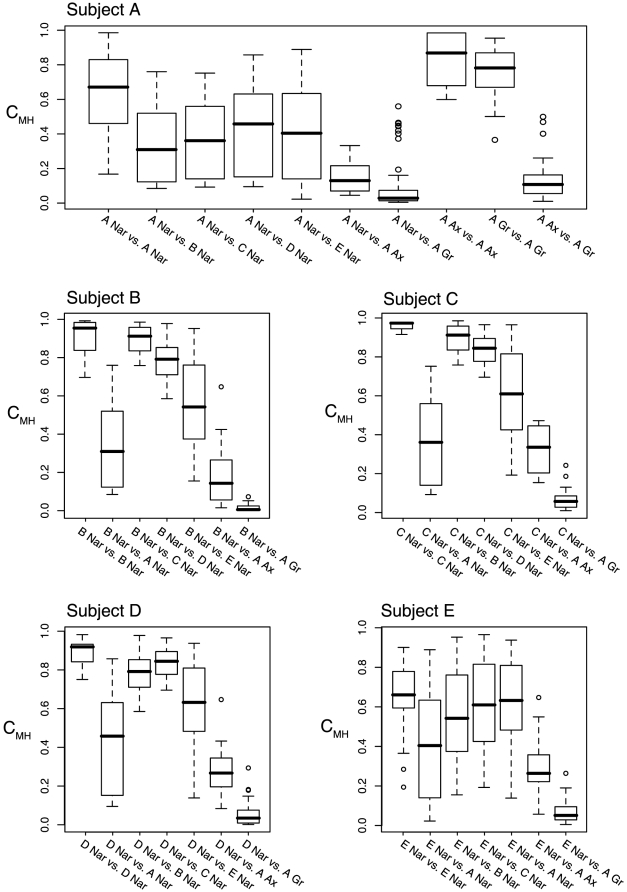
Comparison of community similarity (C_MH_) between specimen types. The similarity of microbiota in all pairwise combinations of specimens obtained from healthy adults was assessed using the abundance-based Morisita-Horn similarity index (C_MH_). Boxplots indicate the spread of C_MH_ values calculated for the indicated comparison. Each chart summarizes data for a study participant. For instance, the first boxplot (“A Nar vs. A Nar”) summarizes data for C_MH_ scores for pairs of Subject A nares samples over time. Statistical significances are reported in Table 2.

Quantitative assessment of similarities between populations (using the Morisita-Horn similarity index) indicated that the nares, axilla and groin harbor distinct and temporally stable microbial communities (Figs. 2 and 3, Table 2). In general, nares samples were more similar to one another than to groin or axilla specimens, even when collected from the same host on the same day (e.g., p<0.001 for intra-subject comparisons of axilla vs. groin, axilla vs. nares, and groin vs. nares in Subject A; Table 2). Interestingly, the axilla and groin microbiotas of Subject A were no more similar to the nares communities of Subject A than to those of Subjects B–E (Fig. 2 and 3, Table 2).

**Table 2:** Community similarity (Morisita-Horn Index) comparisons for healthy adults^1^.

		Subject A			Subject B	Subject C	Subject D	Subject E
		Axilla	Groin	Nares	Nares	Nares	Nares	Nares
Subject A	Axilla	**0.87**	0.11 ***	0.13 ***	0.14 ***	0.34 **	0.27 ***	0.26 ***
	Groin	0.11 ***	**0.78**	0.03 ***	0.01 ***	0.06 ***	0.03 ***	0.05 ***
	Nares	0.13 ***	0.03 ***	**0.67**	0.31 ***	0.36 ***	0.46 ***	0.40 ***
B	Nares	0.14 ***	0.01 ***	0.31 ***	**0.95**	0.91	0.79 ***	0.54
C	Nares	0.34 ***	0.06 ***	0.36 ***	0.91	**0.97**	0.84 *	0.61
D	Nares	0.27 ***	0.03 ***	0.46 ***	0.79 ***	0.84 **	**0.92**	0.63
E	Nares	0.26 ***	0.05 ***	0.40 ***	0.54 ***	0.61 *	0.63 ***	**0.66**

1 Median Morisita-Horn scores for all pairwise combinations of specimen types. Statistical significance assesses whether within-group similarity scores (i.e., axilla specimens vs. axilla specimens), which are in bold, differ significantly from without-group mean similarity scores in the same column (i.e., axilla-specimens vs. groin specimens), measured by Wilcoxon rank-sum test. In this case, the axilla-axilla score of 0.83 differs significantly from the axilla-groin score of 0.14. Significance levels are indicated by

*p<0.05;

**p<0.01;

***p<0.001.

**Figure 4:**
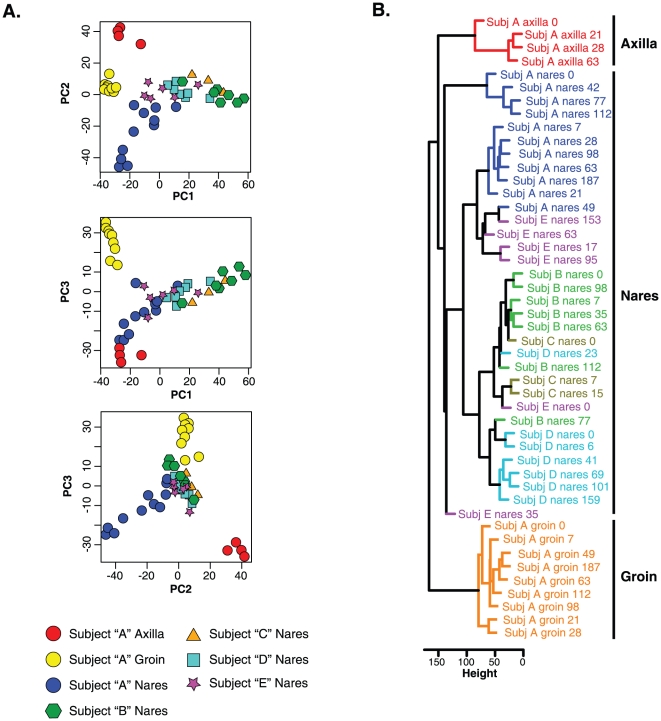
Similarity of microbiotas determined longitudinally for healthy adults. *Panel A. Principal Components Analysis of Microbiotas*. Colors indicate the subject and anatomical location from which longitudinal specimens were obtained. No sampling time-dependent trends were observed in the data, so datapoints are not labeled with respect to time of collection. *Panel B. Hierarchical Clustering*. Colors indicate the subject and anatomical location from which longitudinal specimens were obtained. Leafs are labeled by subject and day of collection. See Materials and Methods for details.

The nares microbiotas of Subjects B, C, and D were indistinguishable from one another, whereas those of Subjects A and E were unique (Figs. 1, 2, and 3). For example, despite some temporal variability, the longitudinal nares samples of Subject A were significantly more similar to each other than to the nares populations of the other subjects (p<0.001 for within-subject to between-subject comparisons; Table 2). Of possible note, Subject A and E are male, Subjects B–D are female, and Subjects A and B and Subjects D and E are co-habitating couples. Thus, cohabitation did not result in convergence of nares microbiotas. Both Principal Components Analysis and hierarchical clustering corroborated these results (Fig. 4) and thereby provide additional evidence of anatomy-specific microbiotas.

**Figure 5:**
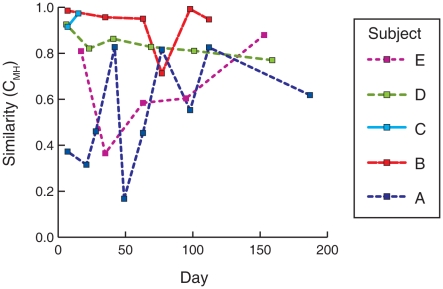
Temporal variation in nares microbiotas of healthy individuals. Each line plots the similarity of a baseline nares specimen to subsequently collected nares specimens from the same individual. Similarity is based on the Morisita-Horn community similarity index.

The temporal variation observed in microbial populations could represent either microbial succession, as newly colonizing microbes displaced other species, or stochastic fluctuations about a fairly constant mean. The latter model, rather than succession, is more consistent with these data because communities remained generally similar to baseline samples over time (Fig. 5).

### Ecology of the Nares Microbiota in Hospitalized Patients

Similar to healthy adults, most of the phylum-level nasal biodiversity observed in the inpatient population was restricted to the Firmicutes (71% of inpatient sequences) and Actinobacteria (20% of inpatient sequences). However, Firmicutes were significantly more abundant in inpatients compared to healthy adults (71% vs. 27% sequence abundance, respectively; Table 1 and Table S2), whereas Actinobacteria were concomitantly less abundant (20% vs. 68% for inpatients vs. healthy).

Differences in the abundances of Firmicutes between inpatients and healthy adults (Table 1) were due primarily to increased abundances of just two species, *S. aureus* and *S. epidermidis*, which together accounted for >50% (10,201/18,080) of the inpatient nares sequences. Similarly, most of the reduction in Actinobacteria in inpatients was associated with significantly diminished abundance of *Propionibacterium acnes* (Table 1).

**Figure 6:**
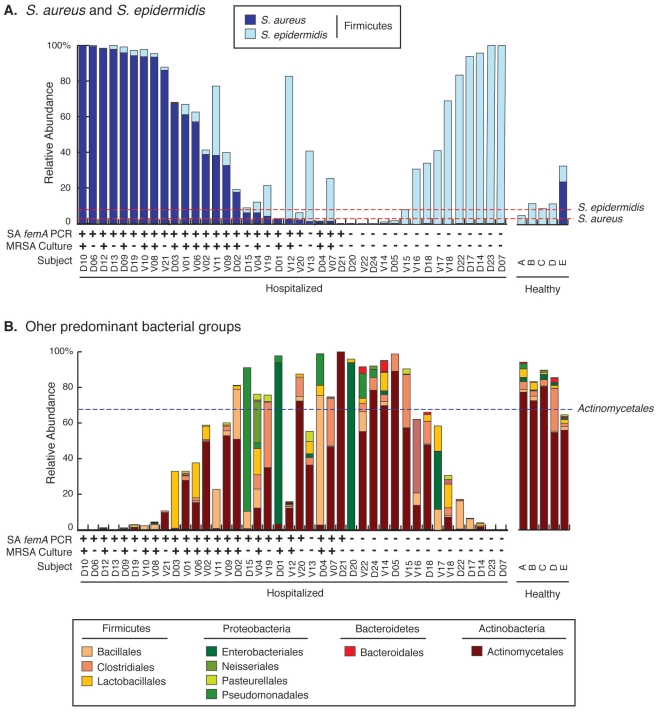
Inter-subject variability in nares microbiota. *Panel A. Comparison of S. aureus and S. epidermidis proportions in rDNA libraries*. Column heights represent relative abundances of particular microbial groups in hospitalized patients and healthy adults. Hospitalized patients are labeled “D” or “V”, based on the hospital ICU at which they were admitted. The dashed lines denote the mean abundances of *S. aureus* and *S. epidermidis* -- 4.8% and 9.3% respectively -- in all nares specimens from healthy adults. rDNA sequences were determined by pyrosequencing. *Panel B. All other bacteria, grouped at taxonomic-order level*. Dashed line represents the mean abundance of Actinomycetales in all nares specimens from healthy adults.

No differences were apparent between the nasal microbiotas of MRSA and MSSA colonized individuals (data not shown), therefore datasets were combined to compare with non-colonized individuals. Among inpatients, *S. aureus* colonization was negatively correlated with *S. epidermidis* abundance (p = 0.004; Table 1). *S. aureus* sequences were the most abundant sequence-types encountered in patients classified as *S. aureus*-carriers by *femA* PCR, whereas *S. epidermidis* sequences were most abundant in the non-carriers (Table 1, Fig. 6). These results both confirm the diagnostic utility of the *femA* PCR assay used to classify patients and indicate that both staphylococcal species can dominate the nares microbiota of certain inpatients. Although several other species, such as *Corynebacterium spp.*, also were less abundant in *S. aureus*-colonized subjects, this study was not sufficiently powered to gain statistical significance. However, the phylum Actinobacteria as a whole was reduced in abundance in *S. aureus* colonized inpatients (p = 0.06).

The dataset presented in Table 1, which pools data from many subjects, masks substantial individual-to-individual variation in nares populations (Fig. 6). The negative correlation between the abundances of *S. aureus* and *S. epidermidis* in *S. aureus* colonized and non-colonized inpatients is readily apparent (Fig. 6A), as is the significant loss of Actinobacteria in inpatients compared with healthy adults (Fig. 6B). In only one patient (V11) were both *S. aureus* and *S. epidermidis* present in relative abundances >25%. Furthermore, a subset of inpatients exhibited elevated levels of Proteobacteria (mainly members of the orders *Enterobacteriales* and *Pseudomonadales*) and Firmicutes other than *S. aureus* and *S. epidermidis*. Thus, blooms in *S. aureus* or *S. epidermidis* were not characteristic of all inpatients, as might be expected given the range of morbidities that likely were exhibited by the inpatient population.

**Figure 7:**
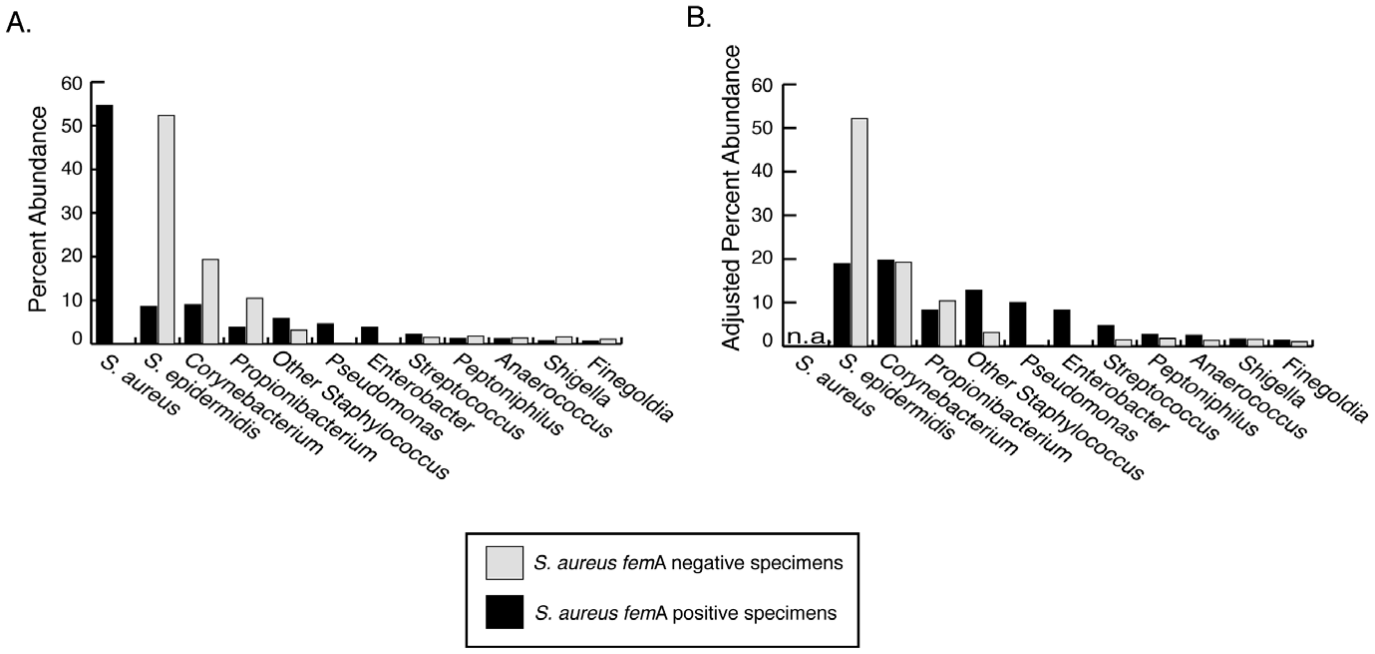
Impact of *S. aureus* sequences on distributions of other nares bacteria. *Panel A*. Percent abundance of top twelve genera. *Panel B*. Percent abundance of top twelve genera adjusted by removal of *S. aureus* from total sequence counts.

In a zero-sum assay such as broad-range PCR, a bloom of one type of microbe would lower the proportions of all other microbes in a sequence library, regardless of whether the bloom actually affected the growth of the other microbes. However, removal of *S. aureus* sequences from the sequence datasets revealed different distributions of microbial groups in *S. aureus* colonized compared with non-colonized individuals (Fig. 7). Of most relevance, the abundance of *S. epidermidis* was greatly reduced relative to most other bacterial taxa in *S. aureus* colonized individuals. This suggests that *S. aureus* may alter the composition of the underlying nares bacterial communities, rather than simply grow without impacting or displacing other microbial communities.

### Reduced Biodiversity in Inpatient Nares Microbiota

**Figure 8:**
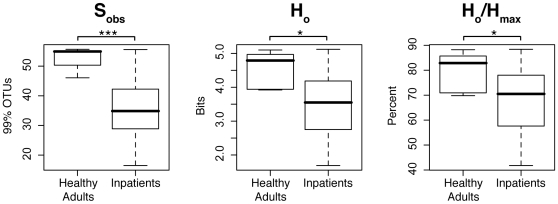
Ecological richness and diversity of nares microbiotas. The top panel presents the distributions of species richness indices (S_obs_) calculated for nares specimens obtained from hospitalized or healthy adults. The middle panel summarizes sample biodiversity (Shannon diversity index H_o_) and the lower panel presents species evenness (H_o_/H_max_. H_o_ = Shannon index; H_max_ = maximum value of H_o_ for a specimen). Significance levels are indicated by * p<0.05; *** p<0.001.

Healthy adults harbored significantly more species-rich and diverse nares microbiotas than did hospitalized individuals (Table 1, Fig. 8). Approximately twice as many species-level microbial groups (defined as 99% OTUs) were identified in healthy adult nares microbiotas as were found in inpatient microbiotas (p<0.001; 245 vs. 112 OTUs, respectively; Table 1) as estimated by the S_chao1_ non-parametric species-richness index (p<0.001; S_obs_: 52 vs. 36 OTUs, respectively; Table 1, Fig. 8). Comparison of Shannon diversity estimates (Table 1, Fig. 8) indicated that inpatient nares microbiotas also were both less diverse (p<0.05) and less even (p<0.05) than healthy microbiotas. Each of these results is consistent with the observed blooms in *S. aureus* and *S. epidermidis*.

### Accuracy of Sequence-based Classification of *Staphylococcus* Species

As described above, sequences were classified and clustered on the basis of BLAST results. Similar results also were obtained when sequences were clustered into 97% and 99% operational taxonomic units (OTUs). We chose to present BLAST-based comparisons, because they tend to mitigate possible over-estimation of OTU-based clusters that can result from sequencing errors, ambiguous alignment, and/or intra-species chimeras [42], [43]. Because the strength of our conclusions with respect to correlations between the abundances of *S. aureus* and other nares-associated species (particularly *S. epidermidis*) is dependent on the accuracy of the rDNA sequence-based microbe identification, we sought to corroborate the results of *S. aureus* and *S. epidermidis* classifications through more detailed sequence analysis.

Because relatively short (<250 nt) pyrosequencing reads might not be as phylogenetically informative as longer sequences, we cloned and Sanger sequenced broad-range amplicons generated from each inpatient sample using the primers 8F and 805R [31], which samples approximately one-half of the 16S rRNA sequence. A total of 3758 Sanger sequences were generated and assigned to taxa in parallel with the pyrosequencing reads discussed above. In general, similar microbial distributions were observed between the two sequencing platforms, despite the use of different PCR primer sets and phylogenetic analysis of partially overlapping sections of the 16S rRNA sequence (Table S2). Indeed, the relative abundances of most taxa varied by less than five-fold between platforms. Of note, *S. aureus* and *S. epidermidis* abundances were qualitatively similar, and thus indicated no systematic bias due to shorter pyrosequencing reads. However, several exceptions were apparent, most notably *Propionibacterium acnes*, which was the 3^rd^ most abundant group in the pyrosequencing dataset, but present at much reduced levels in the Sanger dataset. These differences may have resulted from primer bias or sequence-length dependent ambiguities in classification.

**Figure 9:**
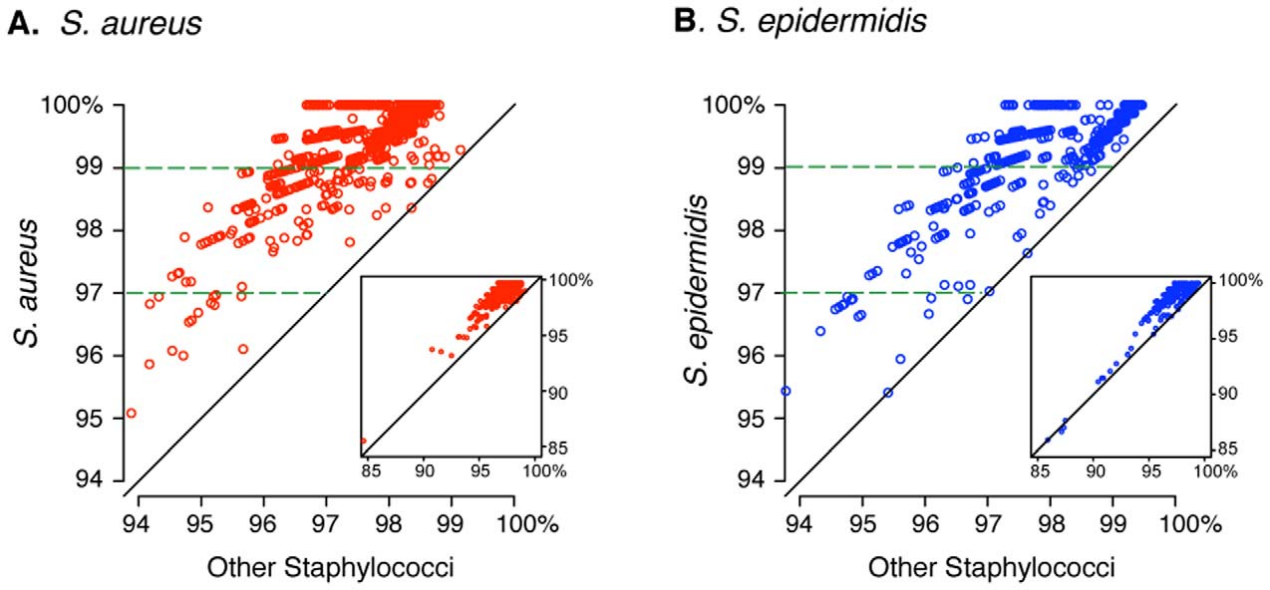
Accuracy of rDNA sequence-based classification of *S. aureus* and *S. epidermidis*. Each nares sequence classified as *S. aureus* or *S. epidermidis* by BLAST query of a highly-curated database (SILVA LTP_S95) was aligned and its pairwise sequence similarity determined in relation to each sequence in a dataset consisting of all LTP_S95 staphylococcal sequences along with other staphylococcal genomic sequences. Maximum percent identity scores were identified both to the predicted species (*S. aureus* or *S. epidermidis*) and to all other staphylococci in the dataset. These two scores were plotted for each nares sequence. Points falling above the diagonal line therefore represent nares sequences that were most closely related to the species predicted by BLAST, whereas points falling below the diagonal suggest mis-classification. The majority of sequences were at least 97% identical to the predicted species, which justifies species-level identification of rRNA sequences. We interpret the low maximum percent identity values obtained for some sequences (i.e.,<97% identity to any staphylococcal species, yet a top BLAST hit of *S. aureus* or *S. epidermidis*) as arising from base-calling or alignment errors inherent in analysis of pyrosequencing reads. *Panel A. Nares sequences classified as S. aureus.* *Panel B. Nares sequences classified as S. epidermidis*.

To further corroborate taxonomic assignments, each presumptive *S. aureus* and *S. epidermidis* sequence was aligned to the SILVA SSURef database (v. 98), then pairwise similarities were calculated against staphylococcal sequences in the Living Tree Project (LTP_S95) rDNA database. For each nares sequence, its highest similarity was noted in comparison to 1) the subset of cognate sequences in the LTP_S95 database (i.e., presumptive *S. aureus* sequences in our dataset vs. *S. aureus* sequences in the database) and 2) all other staphylococcal sequences in the LTP_s95 dataset. Figure 9 presents scatterplots of these two similarity scores plotted for each nares sequence (Panel A for *S. aureus* and Panel B for *S. epidermidis*). In all instances, the nares sequences were most similar to sequences from the predicted species, despite the variability in raw similarity scores. Because the genomic 16S rRNA gene sequences of *S. aureus* and *S. epidermidis* differ by only ca. 1.5%, we were surprised that nares sequences with BLAST %ID or pairwise similarity scores less than 98.5% were most closely related to *S. aureus* or *S. epidermidis*. We attribute this seeming discrepancy to pyrosequencing errors [42], [43] and inaccuracies in sequence alignment, both of which would generally depress all pairwise similarity scores, yet likely preserve the identity of the true nearest neighbor sequence in this type of analysis.

## Discussion

Determination of the biological factors that naturally protect individuals from *S. aureus* colonization may lead to novel strategies for preventing infections. As a first step towards understanding the microbial ecology of *S. aureus* carriage, we have analyzed the nares microbiotas in cohorts of healthy and hospitalized individuals. Patients were classified as *S. aureus* colonized cases or *S. aureus* non-colonized controls, from which we determined and correlated the point-prevalences of *S. aureus* and other microbes in the nares. The nares microbiotas of a small cohort of healthy adults were tracked longitudinally to determine the types and temporal variability of microorganisms that normally inhabit the anterior nares. Although this healthy cohort was not designed to be a control for the inpatient study, it nevertheless provides important context in which to interpret the results of the inpatient point-prevalence survey. For instance, it was critical to first establish whether the anterior nares are inhabited either by a defined, characteristic microbiota or by transient microbial assemblages that result from perpetually changing exposures to bioaerosols, dust particles, water, etc.

Few in-depth longitudinal studies of the human microbiota have been reported to date [44]. Our results indicate that the dominant microorganisms of the axilla, groin, and anterior nares remain relatively constant on the time-scale of weeks to months (Figs. 1 and 2), in the absence of mitigating factors. Although each individual was host to different suites of microorganisms, many commonalities were observed between individuals, such as nasal carriage of *P. acnes*, *S. epidermidis*, and corynebacterial species. Collectively these microorganisms define a pan-microbiota of the human anterior nares as has been reported for other anatomical sites, including the genitourinary tract [45], gut [38], [46], [47], [48], lung [49], and skin [37], [40], [41], [50], [51], [52].

**Figure 10:**
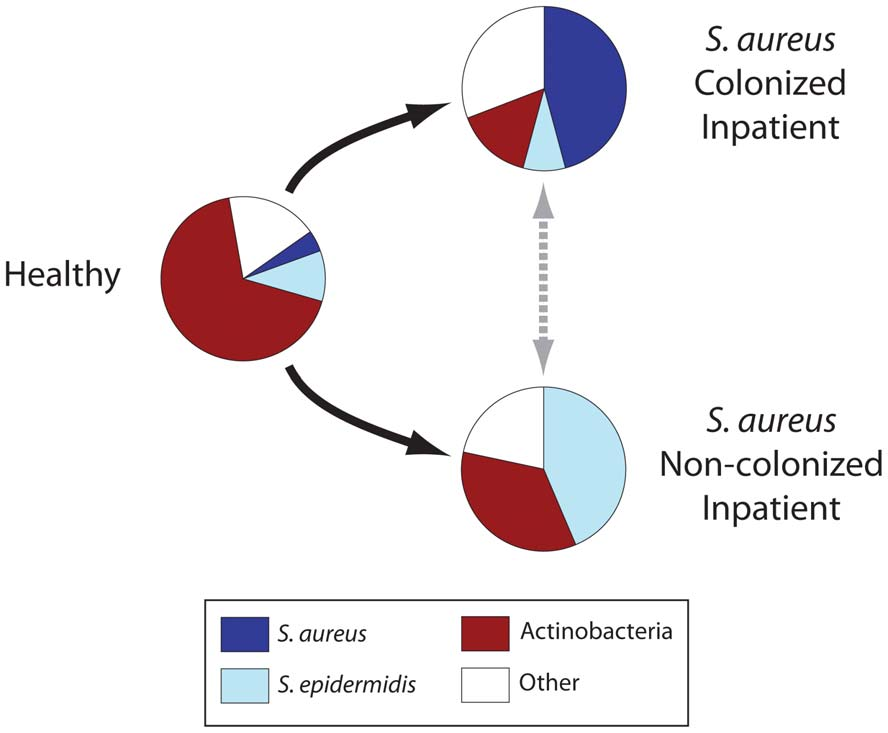
Distinct microbial populations in healthy and hospitalized adults. Pie charts depict average frequencies of dominant microorganisms in the anterior nares of healthy adults and inpatients, classified by *S. aureus* carriage status. Arrows outline possible pathways by which microbial populations develop in hospitalized patients. “Other” represents less abundant taxa, such as Proteobacteria and Firmicutes other than *S. aureus* and *S. epidermidis*.

The nares microbiotas of most hospitalized patients differed substantially from those of the healthy cohort in the kinds and diversities of prevalent microbes. To our knowledge, this is the first characterization of the human nasal microbiota in a clinical context. Our results broadly define three types of microbial populations within the nasal cavity (Fig. 10). First, healthy adults and a subset of inpatients harbored nares communities dominated by Actinobacteria (mainly *Propionibacterium* and *Corynebacterium spp.*), with fewer staphylococci. Second, in the majority of *S. aureus*-colonized inpatients *S. aureus* was the dominant nasal species, with concomitant reductions in the prevalences of Actinobacteria. Third, many *S. aureus* non-colonized patients carried *S. epidermidis* as the dominant species, accompanied by reduced levels of Actinobacteria. Thus in this study, *S. aureus* carriage was negatively associated with a variety of other nares-associated microbial species, most significantly *S. epidermidis* and *P. acnes* (Table 1). These results are consistent with published reports that selected organisms can interfere with *S. aureus* colonization [26], [29]. However, the culture-independent, high-throughput strategy utilized in this study permitted detailed characterization of whole populations of nasal microorganisms, rather than individual species, in relation to *S. aureus* occurrence.

We propose that the underlying morbidities of the inpatient population and/or their exposure to the hospital environment caused a transition from a “healthy” nasal microbiota to either an *S. aureus*- or *S. epidermidis*–dominated microbiota (Fig. 10). Development of an *S. epidermidis*-dominated microbiota may either protect a person from subsequent *S. aureus* colonization or be an intermediate stage in succession to a nasal microbiota dominated by *S. aureus*. In the latter model, loss of commensal species, such as *P. acnes*, rather than gain of *S. epidermidis* could be the key determinant of subsequent *S. aureus* colonization. Because only point-prevalences of microbial groups were determined for inpatients in this study, we could not ascertain whether particular organisms interfere with or promote *S. aureus* colonization; longitudinal studies will shed additional light on the potential for particular microorganisms to inhibit acquisition of *S. aureus* (or *vice versa*). As competing species and strains are identified, the molecular and physiological factors that influence adaptation and competition within the nares environment can be studied in greater depth. For instance, selection for antibiotic resistance and exchange of resistance-encoding genes are expected to be critical factors in nares ecology in the clinical context [53], [54].

The inpatient study population, which was drawn from ICUs, undoubtedly is confounded by myriad co-morbidities, therefore follow-up studies also are necessary to delineate the interplay between specific clinical factors, the nares microbiota, and the risk of *S. aureus* colonization. Nevertheless, similar findings were obtained at two medical centers, suggesting that the results were not unique to a particular patient population or due to methodological bias. Rather, the underlying morbidities of the inpatient study population or exposure to the hospital environment likely account for differences observed between healthy and inpatient cohorts.

Because colonization by *S. aureus* is a significant predisposing factor for subsequent infection, deeper understanding both of colonization and the ecological niche from which infections originate (the nares) could lead to novel prevention strategies. Statistically significant enrichment of particular microbial groups (e.g. *S. epidermidis*) in individuals who are not colonized by *S. aureus* suggests that specific bacterial products interfere with colonization, either by competition for local resources, by antibacterial activity, or by competitive attachment to mucosal sites. Definitive identification and characterization of interfering bacteria or their products may provide candidates for novel medical interventions, both passive and active (e.g., as vaccines or probiotics). For instance, protective microorganisms might be developed for use as probiotic agents within the nasal cavity. Alternatively, the products of *S. aureus*-interfering genes could have potential as topical anti-*S. aureus* agents that block essential binding sites or generate local innate immune activity. The targets of specific inhibitory factors might also suggest candidate antigens for vaccines to prevent *S. aureus* colonization and therefore mitigate the effects of this increasingly prevalent and virulent pathogen.

## Materials and Methods

### Human subjects and sample collection

The study was approved by the Institutional Review Board of the University of Colorado, Boulder (protocols 0807.21 and 1009.35). Prospectively sampled healthy adults provided written informed consent to be enrolled in the study. Specimens collected from hospitalized patients were deidentified of all personal information, so informed consent was not required. Consequently, we were unable to collect clinical or demographic information about the patients beyond MRSA culture results. Nasal swabs were collected through convenience sampling of patients admitted to the intensive care units of two academic medical center hospitals in the Rocky Mountain U.S. region that routinely survey inpatients for nasal MRSA carriage. Specimens were obtained with sterile, dry swabs (Becton, Dickinson and Company), which were rotated five times around the inside of both nostrils while applying constant pressure. Specimens were randomly selected from those collected at the time of admission to intensive care units or ward nursing units, at the time of inter-unit transfer, at time of discharge, or on hospital days 7, 14, 21 etc. Inpatient nasal swabs were used to inoculate MRSA culture plates then promptly preserved in 70% ethanol and stored at −20°C. Nasal swabs from healthy subjects were self-administered in a similar manner, however, right and left nares were sampled separately. Specimens from the axilla and groin were collected by dry swabbing for 5–10 seconds. All swabs were frozen (−20°C) upon collection from healthy individuals.

### MRSA culture

Nasal swabs were cultured on CHROMagar plates (Becton, Dickinson and Company) in a non-CO_2_ incubator and deemed positive if mauve-colored colonies were detected after 18–48 hours incubation. *S. aureus* was confirmed by Gram stain, catalase assay, and/or latex agglutination test.

### DNA extraction

Swab heads were placed in sterile two-ml microcentrifuge tubes with 500 µl of TEN buffer (10 mM Tris-Cl pH 8.0, 1 mM EDTA, 1% NP40 nonionic detergent) and ca. 250 mg of zirconium beads (0.1 mm, Biospec Products Inc, Bartlesville, OK). Specimens were agitated in a Mini Beadbeater-8 (Biospec Products Inc, Bartlesville, OK) on the highest setting for 3 minutes, incubated at 95°C for 10 minutes, vortexed for 30 seconds, and then heated and vortexed a second time. The tubes were either placed on ice or stored at –20°C before PCR processing. Our previous studies indicate that this DNA extraction protocol is robust and efficiently lyses a variety of cell types, including Gram-positive bacteria, mycobacteria, and yeasts [37], [39]. All DNA extraction and PCR steps were performed in a laminar flow hood that was decontaminated by UV light.

### Multiplexed Pyrosequencing

30 µl PCR reactions contained 12 µl 2.5x HotMasterMix (5 PRIME Inc., MD, USA), 0.4 µM 27F-YM3 [55], 0.2 µM barcoded SSU338R [31] and 1 µl mixed-community genomic DNA. A single master PCR cocktail containing all reagents other than the genomic DNA and barcoded SSU338R [32] primers was set up then aliquoted into a 96-well PCR microtitre plate. PCR reactions were performed in duplicate using a protocol of 92°C 15 secs, 52°C 15 secs, and 65°C 45 secs. All samples were initially amplified through 30 cycles and additional cycles were performed if necessary for subsequent sequencing (38 cycles was the maximum).

PCR products were normalized prior to pyrosequencing with the SequalPrepTM Normalization Plate Kit (Invitrogen Inc., CA, USA), following the manufacturer's protocol. Duplicate PCR reactions were pooled prior to normalization. Amplicons were eluted from normalization plates in 30 µl of 10 mM Tris-Cl (pH 8.0). 25 µl aliquots of each amplicon were combined in a pool, which was lyophilized to ∼30 µl. One-half of the pool was electrophoresed through a 1.5% agarose gel in Tris/Acetate/EDTA and product excised with a sterile razor blade under low-wavelength ultraviolet light. DNA was eluted from the gel slice using the MontageTM DNA Gel Extraction Kit (Millipore Corp., MA, USA). This pooled DNA was provided to the Colorado Consortium for Comparative Genomics for pyrosequencing on a 454 Life Sciences GS-FLX instrument.

Barcoded, raw pyrosequencing reads were polished (bases with Q score<20 averaged across window of 5 nts. removed) and deconvoluted using the program *bartab* [32]. 32,341 sequences were excluded from analysis base on the following criteria: 1) trimmed length<175 nt; 2) >0 ambiguous base(s); and/or 3) absence of barcode or forward primer sequence. One specimen, D11, produced only 13 high-quality sequences and was removed from statistical analyses.

### rDNA clone library construction and Sanger sequencing

SSU rRNA genes were amplified from DNA samples by PCR with primers specific for all bacterial SSU rRNA genes: 8F (5′AGAGTTTGATCCTGGCTCAG) and 805R (5′GACTACCAGGGTATCTAAT). Each 30 µl PCR reaction contained 12 µl 2.5x HotMasterMix (5 PRIME Inc., MD, USA), 25 ng of each primer, and 1 µl genomic DNA lysate. rDNA genes were amplified through thirty PCR cycles (92°C 30 sec., 52°C 60 sec., 72°C 90 sec.) and the expected products were inspected using ethidium-bromide-stained agarose gels (Kodak Inc.). PCR controls for each set of samples included extracts from unused, sterile swabs and sterile H_2_O (negative controls).

PCR amplified DNA fragments were excised from agarose gels (1.5% agarose gel in tris-borate EDTA), purified using the QIAquick® gel extraction kit (Qiagen Inc., Valencia, CA), and cloned into the pCR4®-TOPO® vector (TOPO® TA Cloning kit, Invitrogen Corp., Carlsbad, CA). For each clone library, 96 transformants were grown overnight at 37°C in 1.5 ml 2xYT medium using 96-well culture plates. To sequence the inserts of transformants, 20 µl of each overnight culture was mixed with 20 µl of 10 mM Tris-Cl (pH 8.0), heated 10 minutes at 95°C, and centrifuged 10 minutes at 1,360× g in a 96-well plate centrifuge (Eppendorf Inc., Westbury, NY). One microliter of culture supernatant was used as template with vector-specific T7 and T3 primers in a 30 µl PCR reaction (38 cycles as above). Ten microliter of each PCR product were treated with the ExoSap-IT kit (USB Corp, Cleveland, OH) and cycle-sequenced using vector-specific T7 and T3 primers with the Big-Dye Terminator kit (Applied Biosystems, Inc., Foster City, CA). Sequencing was performed in-house on a MegaBACE 1000 (Amersham Biosciences, Piscataway, NJ) automated DNA sequencer. Sequence base calling and contig assembly were performed with the applications phred and phrap [56], [57], as implemented by XplorSeq [58]. Vector and primer sequences were removed along with poor quality flanking nucleotides (Q<20).

### Sequence Analysis

All sequences were aligned to the SILVA SSURef version 98 database using the SINA automated aligner provided by the SILVA web service [36]. Sequences with SILVA quality scores less than 75, including potential chimeric sequences, were removed from the dataset and not subjected to further analysis. Aligned sequences were inserted into the SSURef SILVA guide tree using the parsimony insertion function of ARB [59]. Initial taxonomic assignment of SSU sequences was made by a batch BLAST search of both GenBank and a local database of rRNA sequences extracted from the All-species Living Tree Project database (version LTP_S95; [33]) using the client applications blastcl3 and blastall (NCBI). Taxonomic lineage information was extracted from GenBank records of top BLAST hits and compared to results obtained from the Naïve Bayesian Classifier tool provided by the Ribosomal Database Project [34]. Ambiguities between GenBank and RDP classifications (<0.1% of sequences) were resolved by reference to the results of parsimony insertion into the SILVA guide tree in ARB.

To calculate ecological biodiversity indices, we assigned sequences to OTUs by furthest-neighbor clustering of uncorrected pairwise distances, using the application *sortx* and a distance cutoff of 99% [58]. The completeness of sequencing was assessed by Good's Coverage estimator [60] and estimates of species richness were calculated using the non-parametric estimators ACE (abundance-based coverage estimator [61]) and Chao1 [62] through the program *biodiv* (D.N. Frank, unpublished. http://www.phyloware.com). Sequence distances between presumptive *S. aureus* and *S. epidermidis* 16S rRNA sequences and databased staphylococcal sequences were calculated and compared using the program *xscmpdst* (D.N. Frank, unpublished. http://www.phyloware.com).

### Statistical analyses

All analyses used the R-software package (v.2.0.1; [63]). Differences in the relative abundances of microbial groups between sample types were assessed by the two-tailed Student's t-test for unequal variance (logit-transformation of abundance data did not alter the reported results for untransformed data). Morisita-Horn community similarity indices (C_MH_) were calculated using the vegdist function of the R package “vegan” [64]. Differences in C_MH_ values between categories were tested by Wilcoxon rank-sum test. Hierarchical clustering relied on the R package “cluster” [65] and were generated using both complete and average linkage. Dissimilarity matrices used as input were calculated using either the function daisy (methods “euclidean” and “manhattan” distances) or vegdist (methods “jaccard” and “horn”). Heatmaps used the heatmap.2 function available through the R package “gplots” [66].

### DNA sequence accession numbers

Sequences were deposited in GenBank and assigned the accession numbers HM073607 - HM077276 and HM081990 - HM099519.

## Supporting Information

Table S1 (0.71 MB DOC)

Table S2 (0.61 MB DOC)
