# Supplementary material for: The Human Nasal Microbiota and Staphylococcus aureus Carriage
Source: PLoS One. 2010 May 17;5(5):e10598. doi: 10.1371/journal.pone.0010598 (PMC2871794; doi:10.1371/journal.pone.0010598)
Supplement: Table S1 — (0.71 MB DOC) [file pone.0010598.s001.doc]

**Table S1**. Phylogenetic Distribution of Nares, Groin, and Axilla Microbes in Healthy Adults.

|  | **Location:** | **Nares** | **Groin** | **Axilla** |  |  |  |
| --- | --- | --- | --- | --- | --- | --- | --- |
|  | **Subjects:** | **5** | **1** | **1** |  |  |  |
| **Top Blast Hit^1^** | |  |  |  | **N^2^** | **Accession^3^** | **%ID^4^** |
| **Actinobacteria** | |  |  |  |  |  |  |
|  | *Propionibacterium acnes* | 33.89 | 0.14 | 0.45 | 5566 | AB042288 | 93 - 100 (99) |
|  | *Corynebacterium accolens* | 10.13 | 1 | 0 | 1683 | AJ439346 | 91 - 99 (98) |
|  | *Corynebacterium tuberculostearicum* | 7.39 | 8.07 | 49.22 | 1603 | AJ438050 | 93 - 100 (98) |
|  | *Corynebacterium pseudodiphtheriticum* | 8.41 | 0 | 0 | 1380 | AJ439343 | 92 - 100 (98) |
|  | *Mycobacterium fallax* | 4.83 | 0.05 | 0 | 794 | AF480600 | 90 - 94 (92) |
|  | *Corynebacterium mucifaciens* | 0.47 | 32.23 | 0 | 756 | Y11200 | 92 - 100 (98) |
|  | *Corynebacterium minutissimum* | 0.19 | 18.46 | 0 | 420 | X84678 | 93 - 100 (98) |
|  | *Corynebacterium afermentans subsp. afermentans* | 0.03 | 8.54 | 0 | 185 | X82054 | 92 - 100 (99) |
|  | *Corynebacterium jeikeium* | 0.05 | 6.74 | 0 | 151 | X84250 | 94 - 99 (98) |
|  | *Propionibacterium granulosum* | 0.55 | 0 | 0 | 90 | AJ003057 | 95 - 100 (98) |
|  | *Dermabacter hominis* | 0.03 | 3.99 | 0 | 89 | X91034 | 91 - 98 (96) |
|  | *Corynebacterium thomssenii* | 0.03 | 3.18 | 0 | 72 | AF010474 | 97 - 100 (98) |
|  | *Corynebacterium simulans* | 0.05 | 3.04 | 0 | 72 | AJ012837 | 97 - 100 (99) |
|  | *Corynebacterium variabile* | 0.02 | 2.99 | 0 | 66 | AJ222815 | 92 - 98 (96) |
|  | *Plantibacter auratus* | 0.08 | 2.33 | 0 | 62 | AB012593 | 97 - 100 (99) |
|  | *Propionibacterium avidum* | 0.19 | 1.38 | 0 | 61 | AJ003055 | 97 - 100 (99) |
|  | *Mycobacterium diernhoferi* | 0.11 | 0 | 0 | 18 | AF480599 | 91 - 93 (92) |
|  | *Zimmermannella bifida* | 0.01 | 0.76 | 0 | 18 | AB012595 | 96 - 99 (98) |
|  | *Corynebacterium kroppenstedtii* | 0.1 | 0 | 0 | 17 | Y10077 | 96 - 99 (98) |
|  | *Corynebacterium bovis* | 0.09 | 0 | 0 | 15 | X84444 | 93 - 98 (96) |
|  | *Corynebacterium lipophiloflavum* | 0.05 | 0.33 | 0 | 15 | Y09045 | 95 - 98 (97) |
|  | *Corynebacterium singulare* | 0.01 | 0.57 | 0 | 13 | Y10999 | 93 - 98 (97) |
|  | *Rothia mucilaginosa* | 0.07 | 0 | 0 | 11 | X87758 | 95 - 100 (98) |
|  | *Actinomyces viscosus* | 0.06 | 0 | 0 | 10 | X82453 | 95 - 97 (96) |
|  | *Micrococcus luteus* | 0.06 | 0 | 0 | 10 | AJ536198 | 97 - 100 (98) |
|  | *Brevibacterium paucivorans* | 0.04 | 0.19 | 0 | 10 | AJ251463 | 95 - 99 (97) |
|  | *Pseudonocardia oroxyli* | 0.05 | 0 | 0 | 9 | DQ343154 | 91 - 93 (92) |
|  | *Rothia aeria* | 0.05 | 0 | 0 | 8 | AB071952 | 99 |
|  | *Actinomyces meyeri* | 0.04 | 0 | 0 | 6 | X82451 | 99 |
|  | *Micropruina glycogenica* | 0.01 | 0.19 | 0 | 6 | AB012607 | 92 |
|  | *Actinomyces naeslundii* | 0.04 | 0 | 0 | 6 | X81062 | 91 - 96 (93) |
|  | *Kocuria palustris* | 0.03 | 0 | 0 | 5 | Y16263 | 99 - 100 (99) |
|  | *Corynebacterium urealyticum* | 0.02 | 0.05 | 0 | 4 | X81913 | 91 - 92 (91) |
|  | *Dietzia maris* | 0.02 | 0 | 0 | 4 | X79290 | 90 - 91 (90) |
|  | *Corynebacterium appendicis* | 0 | 0.19 | 0 | 4 | AJ314919 | 98 - 99 (98) |
|  | *Corynebacterium durum* | 0.02 | 0 | 0 | 4 | Z97069 | 96 - 98 (97) |
|  | *Corynebacterium tuscaniense* | 0.02 | 0.05 | 0 | 4 | AY677186 | 97 - 98 (97) |
|  | *Ornithinimicrobium pekingense* | 0.02 | 0 | 0 | 3 | DQ512860 | 97 |
|  | *Corynebacterium matruchotii* | 0.02 | 0 | 0 | 3 | X82065 | 98 - 99 (98) |
|  | *Ornithinimicrobium kibberense* | 0.02 | 0 | 0 | 3 | AY636111 | 97 - 99 (97) |
|  | *Actinomyces georgiae* | 0.02 | 0 | 0 | 3 | X80413 | 96 |
|  | *Microlunatus ginsengisoli* | 0.02 | 0 | 0 | 3 | AB245389 | 95 |
|  | *Actinoplanes digitatis* | 0.02 | 0 | 0 | 3 | AB037000 | 89 |
|  | *Actinomyces odontolyticus* | 0.01 | 0 | 0 | 2 | AJ234040 | 97 - 98 (97) |
|  | *Gordonia terrae* | 0.01 | 0 | 0 | 2 | X79286 | 97 - 99 (98) |
|  | *Rhodococcus fascians* | 0.01 | 0 | 0 | 2 | X79186 | 99 - 100 (99) |
|  | *Kytococcus sedentarius* | 0.01 | 0 | 0 | 2 | X87755 | 93 |
|  | *Micrococcus lylae* | 0.01 | 0 | 0 | 2 | X80750 | 97 - 100 (98) |
|  | *Arthrobacter agilis* | 0.01 | 0 | 0 | 2 | X80748 | 97 - 99 (98) |
|  | *Rothia dentocariosa* | 0.01 | 0 | 0 | 2 | M59055 | 97 |
|  | *Yaniella halotolerans* | 0.01 | 0 | 0 | 2 | AY228479 | 93 - 95 (94) |
|  | *Cryptosporangium minutisporangium* | 0.01 | 0 | 0 | 2 | AB037007 | 93 |
|  | *Corynebacterium propinquum* | 0.01 | 0 | 0.22 | 2 | X84438 | 98 - 99 (98) |
|  | *Agrococcus lahaulensis* | 0.01 | 0 | 0 | 2 | DQ156908 | 97 |
|  | *Scardovia inopinata* | 0.01 | 0 | 0 | 2 | D89332 | 92 - 93 (92) |
|  | *Atopobium parvulum* | 0.01 | 0 | 0 | 2 | X67150 | 97 |
|  | *Acidimicrobium ferrooxidans* | 0.01 | 0 | 0 | 1 | U75647 | 93 |
|  | *Rhodococcus rhodochrous* | 0.01 | 0 | 0 | 1 | X79288 | 93 |
|  | *Agromyces rhizospherae* | 0.01 | 0 | 0 | 1 | AB023357 | 88 |
|  | *Corynebacterium coyleae* | 0.01 | 0 | 0 | 1 | X96497 | 98 |
|  | *Microbacterium terricola* | 0.01 | 0 | 0 | 1 | AB234025 | 98 |
|  | *Corynebacterium xerosis* | 0.01 | 0 | 0 | 1 | X81914 | 94 |
|  | *Geodermatophilus obscurus* | 0.01 | 0 | 0 | 1 | X92356 | 97 |
|  | *Blastococcus saxobsidens* | 0.01 | 0 | 0 | 1 | AJ316571 | 97 |
|  | *Mycobacterium moriokaense* | 0.01 | 0 | 0 | 1 | AJ429044 | 100 |
|  | *Corynebacterium efficiens* | 0.01 | 0 | 0 | 1 | AB055963 | 98 |
|  | *Nocardioides dubius* | 0.01 | 0 | 0 | 1 | AY928902 | 93 |
|  | *Demequina aestuarii* | 0.01 | 0 | 0 | 1 | DQ010160 | 96 |
|  | *Actinoplanes ferrugineus* | 0.01 | 0 | 0 | 1 | AJ277569 | 95 |
|  | *Rathayibacter tritici* | 0.01 | 0 | 0 | 1 | X77438 | 99 |
|  | *Corynebacterium ureicelerivorans* | 0 | 0.05 | 0 | 1 | AM397636 | 97 |
|  | *Arthrobacter psychrophenolicus* | 0.01 | 0 | 0 | 1 | AJ616763 | 98 |
|  | *Corynebacterium glaucum* | 0.01 | 0 | 0 | 1 | AJ431634 | 99 |
|  | *Dietzia papillomatosis* | 0.01 | 0 | 0 | 1 | AY643401 | 96 |
|  | *Curtobacterium citreum* | 0.01 | 0 | 0 | 1 | X77436 | 95 |
|  | *Streptomyces synnematoformans* | 0.01 | 0 | 0 | 1 | EF121313 | 96 |
|  | *Skermania piniformis* | 0.01 | 0 | 0 | 1 | Z35435 | 92 |
|  | *Corynebacterium glutamicum* | 0 | 0.05 | 0 | 1 | AF314192 | 91 |
|  | *Actinomadura rudentiformis* | 0.01 | 0 | 0 | 1 | DQ285420 | 95 |
|  | *Kocuria marina* | 0.01 | 0 | 0 | 1 | AY211385 | 98 |
|  | *Sporichthya polymorpha* | 0.01 | 0 | 0 | 1 | AB025317 | 92 |
|  | *Rhodococcus kunmingensis* | 0.01 | 0 | 0 | 1 | DQ997045 | 92 |
|  | *Saccharomonospora glauca* | 0.01 | 0 | 0 | 1 | Z38003 | 96 |
|  | *Luteococcus peritonei* | 0.01 | 0 | 0 | 1 | AJ132334 | 99 |
|  | *Dermacoccus nishinomiyaensis* | 0.01 | 0 | 0 | 1 | X87757 | 90 |
|  | *Kocuria kristinae* | 0.01 | 0 | 0 | 1 | X80749 | 98 |
|  | *Janibacter anophelis* | 0.01 | 0 | 0 | 1 | AY837752 | 96 |
|  | *Phycicoccus dokdonensis* | 0.01 | 0 | 0 | 1 | EF555583 | 94 |
|  | *Propioniferax innocua* | 0 | 0.05 | 0 | 1 | AF227165 | 95 |
|  | *Actinomyces gerencseriae* | 0.01 | 0 | 0 | 1 | X80414 | 99 |
|  | *Brachybacterium paraconglomeratum* | 0.01 | 0 | 0 | 1 | AJ415377 | 98 |
|  | *Arthrobacter woluwensis* | 0.01 | 0 | 0 | 1 | X93353 | 91 |
|  | *Corynebacterium macginleyi* | 0.01 | 0 | 0 | 1 | AJ439345 | 94 |
|  | *Actinomyces marimammalium* | 0.01 | 0 | 0 | 1 | AJ276405 | 92 |
|  | *Propionibacterium australiense* | 0.01 | 0 | 0 | 1 | AF225962 | 97 |
|  | *Bifidobacterium longum subsp. longum* | 0.01 | 0 | 0 | 1 | M58739 | 97 |
|  | *Gardnerella vaginalis* | 0.01 | 0 | 0 | 1 | M58744 | 100 |
|  | *Parascardovia denticolens* | 0.01 | 0 | 0 | 1 | D89331 | 97 |
| **Bacteroidetes** | |  |  |  |  |  |  |
|  | *Prevotella bivia* | 0.09 | 0.62 | 0 | 27 | L16475 | 89 - 98 (97) |
|  | *Prevotella buccalis* | 0.13 | 0.09 | 0 | 24 | L16476 | 98 - 100 (98) |
|  | *Prevotella corporis* | 0.14 | 0 | 0 | 23 | L16465 | 98 - 100 (99) |
|  | *Prevotella melaninogenica* | 0.1 | 0 | 0 | 17 | AY323525 | 86 - 99 (97) |
|  | *Dysgonomonas gadei* | 0.09 | 0.09 | 0 | 17 | Y18530 | 93 - 94 (93) |
|  | *Prevotella timonensis* | 0.1 | 0 | 0 | 16 | DQ518919 | 98 - 99 (98) |
|  | *Cloacibacterium normanense* | 0.1 | 0 | 0 | 16 | AJ575430 | 90 - 97 (96) |
|  | *Prevotella tannerae* | 0.05 | 0 | 0 | 9 | AJ005634 | 98 - 99 (98) |
|  | *Bacteroides plebeius* | 0.05 | 0 | 0 | 8 | AB200217 | 92 |
|  | *Streptococcus thermophilus* | 0.05 | 0 | 0 | 8 | AY188354 | 98 - 100 (98) |
|  | *Porphyromonas catoniae* | 0.04 | 0 | 0 | 7 | X82823 | 87 - 96 (94) |
|  | *Capnocytophaga leadbetteri* | 0.04 | 0 | 0 | 7 | DQ009623 | 98 - 99 (98) |
|  | *Streptococcus salivarius* | 0.04 | 0 | 0 | 7 | AY188352 | 97 - 100 (98) |
|  | *Capnocytophaga sputigena* | 0.03 | 0 | 0 | 5 | X67609 | 92 - 99 (97) |
|  | *Capnocytophaga gingivalis* | 0.02 | 0 | 0 | 4 | X67608 | 95 - 100 (97) |
|  | *Gramella portivictoriae* | 0.02 | 0 | 0 | 4 | DQ002871 | 91 - 98 (95) |
|  | *Elizabethkingia miricola* | 0.02 | 0 | 0 | 4 | AB071953 | 94 - 99 (97) |
|  | *Hymenobacter soli* | 0.02 | 0 | 0 | 4 | AB251884 | 88 - 95 (91) |
|  | *Prevotella copri* | 0.02 | 0 | 0 | 3 | AB064923 | 97 - 99 (98) |
|  | *Vitellibacter vladivostokensis* | 0.02 | 0 | 0 | 3 | AB071382 | 97 |
|  | *Streptococcus vestibularis* | 0.02 | 0 | 0 | 3 | AY188353 | 97 - 99 (97) |
|  | *Porphyromonas gulae* | 0.01 | 0 | 0 | 2 | AF208290 | 93 |
|  | *Prevotella denticola* | 0.01 | 0 | 0 | 2 | AY323524 | 98 - 99 (98) |
|  | *Prevotella disiens* | 0.01 | 0.05 | 0 | 2 | L16483 | 96 - 99 (97) |
|  | *Chryseobacterium haifense* | 0.01 | 0 | 0 | 2 | EF204450 | 98 |
|  | *Elizabethkingia meningoseptica* | 0.01 | 0 | 0 | 2 | AJ704540 | 97 |
|  | *Terrimonas ferruginea* | 0.01 | 0 | 0 | 2 | AM230484 | 92 - 93 (92) |
|  | *Prevotella maculosa* | 0.01 | 0 | 0 | 1 | EF534314 | 94 |
|  | *Bacteroides thetaiotaomicron* | 0.01 | 0 | 0 | 1 | AE015928 | 99 |
|  | *Prevotella nanceiensis* | 0.01 | 0 | 0 | 1 | AY957555 | 99 |
|  | *Prevotella pleuritidis* | 0.01 | 0 | 0 | 1 | AB278593 | 99 |
|  | *Prevotella veroralis* | 0.01 | 0 | 0 | 1 | L16473 | 95 |
|  | *Bacteroides uniformis* | 0.01 | 0 | 0 | 1 | AB050110 | 100 |
|  | *Prevotella oris* | 0.01 | 0 | 0 | 1 | L16474 | 98 |
|  | *Prevotella oulorum* | 0.01 | 0 | 0 | 1 | L16472 | 100 |
|  | *Capnocytophaga granulosa* | 0.01 | 0 | 0 | 1 | U41347 | 99 |
|  | *Flavobacterium hercynium* | 0.01 | 0 | 0 | 1 | AM265623 | 96 |
|  | *Flavobacterium terrae* | 0.01 | 0 | 0 | 1 | EF117329 | 89 |
|  | *Terrimonas lutea* | 0.01 | 0 | 0 | 1 | AB192292 | 86 |
|  | *Hymenobacter gelipurpurascens* | 0.01 | 0 | 0 | 1 | Y18836 | 87 |
|  | *Dyadobacter koreensis* | 0.01 | 0 | 0 | 1 | EF017660 | 90 |
|  | *Flavisolibacter ginsengiterrae* | 0.01 | 0 | 0 | 1 | AB267476 | 94 |
|  | *Flavisolibacter ginsengisoli* | 0.01 | 0 | 0 | 1 | AB267477 | 88 |
|  | *Sphingobacterium siyangense* | 0.01 | 0 | 0 | 1 | EU046272 | 99 |
|  | *Pontibacter korlensis* | 0.01 | 0 | 0 | 1 | DQ888330 | 94 |
|  | *Pedobacter hartonius* | 0.01 | 0 | 0 | 1 | AM491371 | 97 |
|  | *Hymenobacter ocellatus* | 0.01 | 0 | 0 | 1 | Y18835 | 94 |
| **Cyanobacteria** | |  |  |  |  |  |  |
|  | *Crinalium epipsammum* | 0.07 | 0 | 0 | 11 | AB115964 | 88 - 89 (88) |
|  | *Prochlorococcus marinus subsp. pastoris* | 0.01 | 0 | 0 | 1 | AF180967 | 94 |
|  | *Prochlorothrix hollandica* | 0.01 | 0 | 0 | 1 | AJ007907 | 86 |
| **Deinococcus** | |  |  |  |  |  |  |
|  | *Deinococcus murrayi* | 0.01 | 0 | 0 | 1 | Y13041 | 91 |
|  | *Deinococcus proteolyticus* | 0.01 | 0 | 0 | 1 | Y11331 | 88 |
| **Firmicutes** | |  |  |  |  |  |  |
|  | *Staphylococcus epidermidis* | 9.84 | 0.05 | 41.87 | 1804 | D83363 | 93 - 100 (99) |
|  | *Staphylococcus aureus subsp. aureus* | 4.4 | 0 | 0 | 722 | L36472 | 97 - 100 (99) |
|  | *Peptoniphilus harei* | 3.8 | 0.09 | 0.22 | 626 | Y07839 | 87 - 95 (92) |
|  | *Anaerococcus octavius* | 1.6 | 1.23 | 0 | 289 | Y07841 | 91 - 100 (97) |
|  | *Finegoldia magna* | 1.13 | 1.66 | 0 | 221 | AF542227 | 89 - 100 (99) |
|  | *Dolosigranulum pigrum* | 1.29 | 0 | 0 | 212 | X70907 | 91 - 98 (97) |
|  | *Anaerococcus vaginalis* | 0.86 | 0.33 | 0 | 148 | AF542229 | 88 - 98 (91) |
|  | *Staphylococcus caprae* | 0.47 | 0.05 | 6.24 | 106 | AB009935 | 96 - 100 (99) |
|  | *Streptococcus mitis* | 0.52 | 0 | 0 | 85 | AF003929 | 91 - 100 (98) |
|  | *Staphylococcus saccharolyticus* | 0.4 | 0 | 0 | 65 | L37602 | 98 - 100 (99) |
|  | *Megasphaera micronuciformis* | 0.21 | 0 | 0 | 35 | AF473834 | 90 - 99 (92) |
|  | *Dialister propionicifaciens* | 0.17 | 0 | 0 | 28 | AY850119 | 99 - 100 (99) |
|  | *Bacillus siralis* | 0.13 | 0 | 0 | 21 | AF071856 | 88 - 95 (93) |
|  | *Streptococcus infantis* | 0.12 | 0 | 0 | 20 | AY485603 | 96 - 99 (97) |
|  | *Streptococcus sanguinis* | 0.11 | 0 | 0 | 18 | AF003928 | 95 - 99 (97) |
|  | *Streptococcus oralis* | 0.09 | 0 | 0 | 14 | AY485602 | 98 - 99 (98) |
|  | *Staphylococcus warneri* | 0.05 | 0 | 0.67 | 12 | L37603 | 95 - 100 (99) |
|  | *Aerococcus urinaeequi* | 0.07 | 0 | 0 | 12 | D87677 | 98 - 99 (98) |
|  | *Streptococcus australis* | 0.07 | 0 | 0 | 11 | AY485604 | 97 - 99 (98) |
|  | *Staphylococcus auricularis* | 0.05 | 0 | 0 | 9 | D83358 | 98 - 100 (98) |
|  | *Veillonella parvula* | 0.05 | 0 | 0 | 9 | AY995767 | 97 - 99 (97) |
|  | *Granulicatella adiacens* | 0.05 | 0 | 0 | 8 | D50540 | 95 - 99 (97) |
|  | *Lactobacillus fornicalis* | 0.05 | 0 | 0 | 8 | Y18654 | 95 - 100 (98) |
|  | *Streptococcus peroris* | 0.05 | 0 | 0 | 8 | AB008314 | 96 - 97 (96) |
|  | *Faecalibacterium prausnitzii* | 0.04 | 0 | 0 | 7 | AJ413954 | 94 - 97 (95) |
|  | *Veillonella ratti* | 0.04 | 0 | 0 | 7 | AY355138 | 92 - 93 (92) |
|  | *Selenomonas noxia* | 0.04 | 0 | 0 | 6 | AF287799 | 95 - 100 (97) |
|  | *Veillonella rogosae* | 0.04 | 0 | 0 | 6 | EF108443 | 93 - 99 (97) |
|  | *Veillonella atypica* | 0.04 | 0 | 0 | 6 | AF439641 | 98 - 100 (99) |
|  | *Veillonella dispar* | 0.04 | 0 | 0 | 6 | AF439639 | 97 - 99 (98) |
|  | *Staphylococcus lugdunensis* | 0.03 | 0 | 0 | 5 | AB009941 | 97 - 99 (97) |
|  | *Staphylococcus hominis subsp. novobiosepticus* | 0.03 | 0 | 0 | 5 | AB233326 | 99 - 100 (99) |
|  | *Staphylococcus pasteuri* | 0.03 | 0 | 0 | 5 | AB009944 | 96 - 100 (98) |
|  | *Staphylococcus hominis subsp. hominis* | 0.03 | 0 | 0 | 5 | X66101 | 99 |
|  | *Veillonella rodentium* | 0.03 | 0 | 0 | 5 | AY514996 | 98 - 99 (98) |
|  | *Selenomonas sputigena* | 0.03 | 0 | 0 | 5 | AF287793 | 96 - 98 (96) |
|  | *Lactobacillus crispatus* | 0.03 | 0 | 0 | 5 | AF257097 | 99 - 100 (99) |
|  | *Streptococcus pseudopneumoniae* | 0.03 | 0 | 0 | 5 | AY612844 | 93 - 99 (96) |
|  | *Eremococcus coleocola* | 0 | 0.19 | 0 | 4 | Y17780 | 90 - 97 (93) |
|  | *Streptococcus parasanguinis* | 0.02 | 0 | 0 | 4 | AF003933 | 96 - 98 (96) |
|  | *Gemella haemolysans* | 0.02 | 0 | 0 | 3 | L14326 | 98 - 99 (98) |
|  | *Clostridium lituseburense* | 0.02 | 0 | 0 | 3 | M59107 | 96 |
|  | *Ruminococcus obeum* | 0.01 | 0.05 | 0 | 3 | X85101 | 94 - 99 (95) |
|  | *Selenomonas infelix* | 0.02 | 0 | 0 | 3 | AF287802 | 98 |
|  | *Veillonella denticariosi* | 0.02 | 0 | 0 | 3 | EF185167 | 98 |
|  | *Atopostipes suicloacalis* | 0.02 | 0 | 0 | 3 | AF445248 | 92 - 97 (93) |
|  | *Lactobacillus gasseri* | 0.02 | 0 | 0 | 3 | AF519171 | 96 - 100 (98) |
|  | *Planococcus donghaensis* | 0.01 | 0 | 0 | 2 | EF079063 | 98 - 99 (98) |
|  | *Staphylococcus cohnii subsp. urealyticus* | 0.01 | 0 | 0 | 2 | AB009936 | 98 - 99 (98) |
|  | *Staphylococcus saprophyticus subsp. saprophyticus* | 0.01 | 0 | 0 | 2 | AP008934 | 99 - 100 (99) |
|  | *Roseburia intestinalis* | 0.01 | 0 | 0 | 2 | AJ312385 | 99 |
|  | *Veillonella caviae* | 0.01 | 0 | 0 | 2 | AY355140 | 92 - 98 (95) |
|  | *Ignavigranum ruoffiae* | 0 | 0.09 | 0 | 2 | Y16426 | 88 - 89 (88) |
|  | *Vagococcus fluvialis* | 0.01 | 0 | 0 | 2 | Y18098 | 98 - 99 (98) |
|  | *Lactobacillus iners* | 0.01 | 0 | 0 | 2 | Y16329 | 99 |
|  | *Streptococcus cristatus* | 0.01 | 0 | 0 | 2 | AY188347 | 97 - 99 (98) |
|  | *Streptococcus intermedius* | 0.01 | 0 | 0 | 2 | AF104671 | 99 - 100 (99) |
|  | *Bacillus subtilis subsp. subtilis* | 0.01 | 0 | 0 | 1 | AJ276351 | 99 |
|  | *Geobacillus tepidamans* | 0.01 | 0 | 0 | 1 | AY563003 | 98 |
|  | *Gemella morbillorum* | 0.01 | 0 | 0 | 1 | L14327 | 100 |
|  | *Gemella sanguinis* | 0.01 | 0 | 0 | 1 | Y13364 | 99 |
|  | *Exiguobacterium artemiae* | 0.01 | 0 | 0 | 1 | AM072763 | 100 |
|  | *Jeotgalicoccus halotolerans* | 0.01 | 0 | 0 | 1 | AY028925 | 92 |
|  | *Ruminococcus luti* | 0.01 | 0 | 0 | 1 | AJ133124 | 97 |
|  | *Clostridium nexile* | 0 | 0.05 | 0 | 1 | X73443 | 97 |
|  | *Clostridium hiranonis* | 0.01 | 0 | 0 | 1 | AB023970 | 99 |
|  | *Clostridium bifermentans* | 0.01 | 0 | 0 | 1 | AB075769 | 91 |
|  | *Thermohalobacter berrensis* | 0.01 | 0 | 0 | 1 | AF113543 | 99 |
|  | *Clostridium saccharoperbutylacetonicum* | 0.01 | 0 | 0 | 1 | U16122 | 93 |
|  | *Clostridium methoxybenzovorans* | 0.01 | 0 | 0 | 1 | AF067965 | 95 |
|  | *Anaerococcus tetradius* | 0.01 | 0 | 0 | 1 | AF542234 | 96 |
|  | *Anaerococcus prevotii* | 0.01 | 0 | 0 | 1 | AF542232 | 95 |
|  | *Peptoniphilus lacrimalis* | 0.01 | 0 | 0 | 1 | AF542230 | 89 |
|  | *Mogibacterium vescum* | 0.01 | 0 | 0 | 1 | AB021702 | 88 |
|  | *Anaerococcus hydrogenalis* | 0 | 0.05 | 0 | 1 | D14140 | 97 |
|  | *Parvimonas micra* | 0.01 | 0 | 0 | 1 | AY323523 | 100 |
|  | *Eubacterium eligens* | 0.01 | 0 | 0 | 1 | L34420 | 94 |
|  | *Moryella indoligenes* | 0.01 | 0 | 0 | 1 | DQ377947 | 95 |
|  | *Butyrivibrio fibrisolvens* | 0.01 | 0 | 0 | 1 | U41172 | 92 |
|  | *Oribacterium sinus* | 0.01 | 0 | 0 | 1 | AY323228 | 90 |
|  | *Ruminococcus callidus* | 0.01 | 0 | 0 | 1 | L76596 | 93 |
|  | *Ruminococcus gnavus* | 0.01 | 0 | 0 | 1 | X94967 | 98 |
|  | *Dendrosporobacter quercicolus* | 0.01 | 0 | 0 | 1 | AJ010962 | 89 |
|  | *Catenibacterium mitsuokai* | 0.01 | 0 | 0 | 1 | AB030224 | 94 |
|  | *Eubacterium biforme* | 0.01 | 0 | 0 | 1 | M59230 | 98 |
|  | *Lactobacillus plantarum subsp. plantarum* | 0 | 0.05 | 0 | 1 | AJ965482 | 100 |
|  | *Lactobacillus delbrueckii subsp. bulgaricus* | 0.01 | 0 | 0 | 1 | CR954253 | 100 |
|  | *Leuconostoc pseudomesenteroides* | 0.01 | 0 | 0 | 1 | AB023237 | 97 |
|  | *Lactococcus lactis subsp. cremoris* | 0.01 | 0 | 0 | 1 | AB100802 | 99 |
|  | *Streptococcus anginosus* | 0.01 | 0 | 0 | 1 | AF104678 | 99 |
|  | *Streptococcus infantarius subsp. coli* | 0.01 | 0 | 0 | 1 | AF429763 | 99 |
| **Fusobacteria** | |  |  |  |  |  |  |
|  | *Fusobacterium nucleatum subsp. vincentii* | 0.07 | 0 | 0 | 12 | AABF01000111 | 97 - 99 (98) |
|  | *Leptotrichia wadei* | 0.07 | 0 | 0 | 11 | AY029802 | 94 - 99 (97) |
|  | *Fusobacterium canifelinum* | 0.03 | 0 | 0 | 5 | AY162221 | 96 - 98 (97) |
|  | *Fusobacterium nucleatum subsp. animalis* | 0.02 | 0 | 0 | 4 | X55404 | 98 - 99 (98) |
|  | *Fusobacterium periodonticum* | 0.01 | 0 | 0 | 1 | X55405 | 97 |
|  | *Leptotrichia goodfellowii* | 0.01 | 0 | 0 | 1 | AY029807 | 86 |
| **Proteobacteria** | |  |  |  |  |  |  |
|  | *Moraxella catarrhalis* | 0.56 | 0 | 0 | 92 | AF005185 | 97 - 100 (99) |
|  | *Microvirgula aerodenitrificans* | 0.43 | 0 | 0 | 70 | U89333 | 86 - 94 (90) |
|  | *Salmonella enterica subsp. diarizonae* | 0.3 | 0.05 | 0 | 51 | EU014688 | 96 - 99 (98) |
|  | *Raoultella planticola* | 0.3 | 0 | 0 | 49 | AF129443 | 98 - 100 (99) |
|  | *Bacteroides ureolyticus* | 0.22 | 0 | 0 | 36 | L04321 | 97 - 98 (97) |
|  | *Haemophilus parainfluenzae* | 0.22 | 0 | 0 | 36 | AY362908 | 94 - 98 (96) |
|  | *Salmonella enterica subsp. arizonae* | 0.19 | 0.05 | 0 | 33 | AF008580 | 97 - 99 (98) |
|  | *Pannonibacter phragmitetus* | 0.16 | 0 | 0 | 27 | AJ400704 | 94 - 96 (95) |
|  | *Neisseria flavescens* | 0.11 | 0 | 0 | 18 | L06168 | 94 - 99 (97) |
|  | *Enterobacter ludwigii* | 0.09 | 0 | 0.22 | 16 | AJ853891 | 97 - 100 (99) |
|  | *Propionivibrio dicarboxylicus* | 0.09 | 0 | 0 | 14 | Y17601 | 97 - 98 (97) |
|  | *Enhydrobacter aerosaccus* | 0.09 | 0 | 0 | 14 | AJ550856 | 92 - 99 (97) |
|  | *Sphingomonas faeni* | 0.07 | 0 | 0 | 12 | AJ429239 | 96 - 100 (98) |
|  | *Aquabacterium commune* | 0.05 | 0 | 0 | 8 | AF035054 | 97 - 98 (97) |
|  | *Achromobacter xylosoxidans* | 0.01 | 0.28 | 0 | 8 | Y14908 | 95 - 97 (96) |
|  | *Neisseria elongata subsp. glycolytica* | 0.04 | 0 | 0 | 7 | AY167422 | 99 - 100 (99) |
|  | *Shigella sonnei* | 0.03 | 0.09 | 0 | 7 | X80726 | 93 - 100 (98) |
|  | *Pasteurella mairii* | 0.04 | 0 | 0 | 7 | AY362923 | 95 - 97 (96) |
|  | *Rubellimicrobium thermophilum* | 0.04 | 0 | 0 | 6 | AJ844281 | 90 - 93 (91) |
|  | *Acidisphaera rubrifaciens* | 0.04 | 0 | 0 | 6 | D86512 | 96 - 97 (96) |
|  | *Burkholderia ambifaria* | 0.04 | 0 | 0 | 6 | AF043302 | 100 |
|  | *Salmonella enterica subsp. salamae* | 0.04 | 0 | 0 | 6 | EU014685 | 96 - 99 (97) |
|  | *Citrobacter farmeri* | 0.03 | 0.05 | 0 | 6 | AF025371 | 97 - 99 (98) |
|  | *Nitrosomonas eutropha* | 0.03 | 0 | 0 | 5 | AY123795 | 95 - 96 (95) |
|  | *Citrobacter murliniae* | 0.02 | 0.05 | 0.22 | 5 | AF025369 | 99 - 100 (99) |
|  | *Serratia fonticola* | 0.03 | 0 | 0 | 5 | AJ233429 | 97 - 98 (97) |
|  | *Yersinia enterocolitica subsp. enterocolitica* | 0.02 | 0.05 | 0 | 5 | AF366378 | 97 - 98 (97) |
|  | *Roseicyclus mahoneyensis* | 0.02 | 0 | 0 | 4 | AJ315682 | 96 |
|  | *Rhodobacter veldkampii* | 0.02 | 0 | 0 | 4 | D16421 | 99 |
|  | *Sphingomonas yanoikuyae* | 0.02 | 0 | 0 | 4 | D13728 | 97 - 99 (98) |
|  | *Acinetobacter radioresistens* | 0.02 | 0 | 0 | 4 | X81666 | 97 - 98 (97) |
|  | *Acinetobacter lwoffii* | 0.02 | 0 | 0 | 4 | X81665 | 100 |
|  | *Methylobacterium iners* | 0.02 | 0 | 0 | 3 | EF174497 | 94 - 95 (94) |
|  | *Caenispirillum bisanense* | 0.02 | 0 | 0 | 3 | EF100694 | 95 - 98 (96) |
|  | *Massilia aurea* | 0.02 | 0 | 0 | 3 | AM231588 | 99 - 100 (99) |
|  | *Delftia tsuruhatensis* | 0.02 | 0 | 0 | 3 | AB075017 | 99 - 100 (99) |
|  | *Neisseria lactamica* | 0.02 | 0 | 0 | 3 | AJ239286 | 93 |
|  | *Natronocella acetinitrilica* | 0.02 | 0 | 0 | 3 | EF103128 | 96 - 97 (96) |
|  | *Enterobacter kobei* | 0.02 | 0 | 0 | 3 | AJ508301 | 99 |
|  | *Pseudomonas psychrophila* | 0.02 | 0 | 0 | 3 | AB041885 | 97 - 100 (99) |
|  | *Roseomonas aerilata* | 0.01 | 0 | 0 | 2 | EF661571 | 97 |
|  | *Sphingomonas phyllosphaerae* | 0.01 | 0 | 0 | 2 | AY453855 | 94 |
|  | *Sphingomonas herbicidovorans* | 0.01 | 0 | 0 | 2 | AB022428 | 98 |
|  | *Erythrobacter vulgaris* | 0.01 | 0 | 0 | 2 | AY706935 | 100 |
|  | *Herbaspirillum seropedicae* | 0.01 | 0 | 0 | 2 | Y10146 | 98 |
|  | *Pelomonas aquatica* | 0.01 | 0 | 0 | 2 | AM501435 | 95 - 99 (97) |
|  | *Pseudomonas balearica* | 0 | 0 | 0.45 | 2 | U26418 | 99 |
|  | *Pseudomonas aeruginosa* | 0.01 | 0 | 0 | 2 | X06684 | 98 |
|  | *Pseudomonas stutzeri* | 0.01 | 0 | 0 | 2 | AF094748 | 98 |
|  | *Sulfurivirga caldicuralii* | 0.01 | 0 | 0 | 2 | AB245479 | 93 |
|  | *Brevundimonas vesicularis* | 0.01 | 0 | 0 | 1 | AJ227780 | 99 |
|  | *Phenylobacterium lituiforme* | 0.01 | 0 | 0 | 1 | AY534887 | 96 |
|  | *Afipia massiliensis* | 0.01 | 0 | 0 | 1 | AY029562 | 97 |
|  | *Blastochloris sulfoviridis* | 0.01 | 0 | 0 | 1 | D86514 | 94 |
|  | *Devosia riboflavina* | 0.01 | 0 | 0 | 1 | AJ549086 | 99 |
|  | *Rhizobium huautlense* | 0.01 | 0 | 0 | 1 | AF025852 | 97 |
|  | *Methylobacterium aerolatum* | 0.01 | 0 | 0 | 1 | EF174498 | 94 |
|  | *Bradyrhizobium elkanii* | 0.01 | 0 | 0 | 1 | U35000 | 98 |
|  | *Shinella granuli* | 0.01 | 0 | 0 | 1 | AB187585 | 96 |
|  | *Octadecabacter arcticus* | 0.01 | 0 | 0 | 1 | U73725 | 92 |
|  | *Paracoccus marcusii* | 0.01 | 0 | 0 | 1 | Y12703 | 100 |
|  | *Gluconobacter frateurii* | 0.01 | 0 | 0 | 1 | X82290 | 98 |
|  | *Muricoccus roseus* | 0.01 | 0 | 0 | 1 | AJ488505 | 94 |
|  | *Novosphingobium pentaromativorans* | 0.01 | 0 | 0 | 1 | AF502400 | 97 |
|  | *Sphingomonas ursincola* | 0.01 | 0 | 0 | 1 | AB024289 | 99 |
|  | *Sphingomonas paucimobilis* | 0.01 | 0 | 0 | 1 | AM237364 | 96 |
|  | *Sphingopyxis chilensis* | 0.01 | 0 | 0 | 1 | AF367204 | 98 |
|  | *Sphingomonas chlorophenolica* | 0.01 | 0 | 0 | 1 | X87161 | 98 |
|  | *Duganella zoogloeoides* | 0.01 | 0 | 0 | 1 | D14256 | 99 |
|  | *Undibacterium pigrum* | 0.01 | 0 | 0 | 1 | AM397630 | 94 |
|  | *Herbaspirillum huttiense* | 0.01 | 0 | 0 | 1 | AB021366 | 99 |
|  | *Ralstonia mannitolilytica* | 0.01 | 0 | 0 | 1 | AJ270258 | 98 |
|  | *Caenimonas koreensis* | 0.01 | 0 | 0 | 1 | DQ349098 | 92 |
|  | *Burkholderia phytofirmans* | 0.01 | 0 | 0 | 1 | AY497470 | 95 |
|  | *Tetrathiobacter mimigardefordensis* | 0 | 0.05 | 0 | 1 | AY880023 | 96 |
|  | *Leptothrix mobilis* | 0.01 | 0 | 0 | 1 | X97071 | 98 |
|  | *Ralstonia pickettii* | 0.01 | 0 | 0 | 1 | AY741342 | 99 |
|  | *Massilia brevitalea* | 0.01 | 0 | 0 | 1 | EF546777 | 98 |
|  | *Burkholderia fungorum* | 0 | 0 | 0.22 | 1 | AF215705 | 97 |
|  | *Massilia aerilata* | 0.01 | 0 | 0 | 1 | EF688526 | 95 |
|  | *Schlegelella aquatica* | 0.01 | 0 | 0 | 1 | DQ417336 | 100 |
|  | *Pelomonas puraquae* | 0.01 | 0 | 0 | 1 | AM501439 | 98 |
|  | *Rhodocyclus tenuis* | 0.01 | 0 | 0 | 1 | D16208 | 86 |
|  | *Dechloromonas agitata* | 0.01 | 0 | 0 | 1 | AF047462 | 99 |
|  | *Syntrophobacter wolinii* | 0.01 | 0 | 0 | 1 | X70905 | 93 |
|  | *Campylobacter gracilis* | 0.01 | 0 | 0 | 1 | DQ174168 | 99 |
|  | *Campylobacter showae* | 0.01 | 0 | 0 | 1 | DQ174155 | 97 |
|  | *Saccharophagus degradans* | 0.01 | 0 | 0 | 1 | AF055269 | 89 |
|  | *Lamprocystis roseopersicina* | 0.01 | 0 | 0 | 1 | AJ006063 | 96 |
|  | *Erwinia persicina* | 0.01 | 0 | 0 | 1 | U80205 | 98 |
|  | *Arsenophonus nasoniae* | 0.01 | 0 | 0 | 1 | AY264674 | 98 |
|  | *Salmonella enterica subsp. indica* | 0.01 | 0 | 0 | 1 | EU014680 | 98 |
|  | *Actinobacillus equuli subsp. haemolyticus* | 0.01 | 0 | 0 | 1 | AF247716 | 96 |
|  | *Haemophilus pittmaniae* | 0.01 | 0 | 0 | 1 | AJ290755 | 100 |
|  | *Pasteurella aerogenes* | 0.01 | 0 | 0 | 1 | U66491 | 97 |
|  | *Pasteurella caballi* | 0.01 | 0 | 0 | 1 | AY362918 | 96 |
|  | *Aggregatibacter actinomycetemcomitans* | 0.01 | 0 | 0 | 1 | M75039 | 95 |
|  | *Acinetobacter haemolyticus* | 0.01 | 0 | 0 | 1 | X81662 | 99 |
|  | *Pseudomonas pohangensis* | 0.01 | 0 | 0 | 1 | DQ339144 | 100 |
|  | *Acinetobacter johnsonii* | 0.01 | 0 | 0 | 1 | Z93440 | 98 |
|  | *Moraxella lacunata* | 0.01 | 0 | 0 | 1 | D64049 | 97 |
|  | *Stenotrophomonas maltophilia* | 0.01 | 0 | 0 | 1 | AB294553 | 100 |
|  | *Stenotrophomonas koreensis* | 0.01 | 0 | 0 | 1 | AB166885 | 98 |
|  | *Thermomonas hydrothermalis* | 0.01 | 0 | 0 | 1 | AF542054 | 93 |
|  | *Pseudomonas beteli* | 0.01 | 0 | 0 | 1 | AB021406 | 99 |
| **Tenericutes** | |  |  |  |  |  |  |
|  | *Ureaplasma parvum* | 0.07 | 0 | 0 | 11 | AF073456 | 99 - 100 (99) |
|  | **Total** | 100% | 100% | 100% | 18967 |  |  |
|  | **N^2^** | 16411 | 2107 | 449 | 18967 |  |  |
|  |  |  |  |  |  |  |  |
| ^1^ Identity of microorganisms inferred from highest bit-score in BLAST query. Hits with Blast %IDs < 97 are named only to the genus level. | | | | | | | |
| ^2^ Number of sequences analyzed for group. | |  |  |  |  |  |  |
| ^3^ GenBank accession number for top Blast hit. | |  |  |  |  |  |  |
| ^4^ Mean Blast percent identity score for top Blast hit | |  |  |  |  |  |  |
| ^5^ Percent of sequences classified as species/genus for *fem*A-based category. Columns sum to 100% | | | | | | |  |
| ^6^ Lower abundance sequences (< 10% of total) were omitted to simplify the table | | | | |  |  |  |
