# Supplementary material for: The Human Nasal Microbiota and Staphylococcus aureus Carriage
Source: PLoS One. 2010 May 17;5(5):e10598. doi: 10.1371/journal.pone.0010598 (PMC2871794; doi:10.1371/journal.pone.0010598)
Supplement: Table S2 — (0.61 MB DOC) [file pone.0010598.s002.doc]

**Table S2.** Phylogenetic Distribution of Nares Microbes in Hospital Inpatients

|  | **Platform^1^:** | **Pyrosequencing** | | | **Sanger** | | |  |  |  |
| --- | --- | --- | --- | --- | --- | --- | --- | --- | --- | --- |
|  | ***femA* PCR^2^:** | **Neg** | **Pos** | **N^5^** | **Neg** | **Pos** | **N^5^** |  |  |  |
| **Top Blast Hit^4^** | |  |  |  |  |  |  | **Accession^6^** | **%ID^7^** | **Fold** |
| **Actinobacteria** | |  |  |  |  |  |  |  |  |  |
|  | *Corynebacterium accolens* | 5.57 | 5.19 | 726 | 7.50 | 5.67 | 241 | AJ439346 | 94 - 100 (98) | 0.8 |
|  | *Propionibacterium acnes* | 12.15 | 3.38 | 821 | 0.21 | 0.04 | 4 | AB042288 | 92 - 100 (98) | 53.8 |
|  | *Corynebacterium tuberculostearicum* | 5.69 | 1.01 | 276 | 5.55 | 1.71 | 123 | AJ438050 | 93 - 100 (99) | 0.6 |
|  | *Corynebacterium tuscaniense* | 4.96 | 0.00 | 246 | 0.55 | 0.00 | 7 | AY677186 | 93 - 98 (97) | 9.2 |
|  | *Corynebacterium propinquum* | 0.24 | 1.65 | 148 | 0.48 | 3.10 | 83 | X84438 | 96 - 99 (98) | 0.5 |
|  | *Corynebacterium macginleyi* | 3.21 | 0.15 | 183 | 2.53 | 0.23 | 42 | AJ439345 | 94 - 100 (98) | 1.1 |
|  | *Corynebacterium pseudodiphtheriticum* | 0.00 | 1.92 | 164 | 0.13 | 1.99 | 38 | AJ439343 | 95 - 100 (98) | 1.1 |
|  | *Propionibacterium granulosum* | 0.22 | 0.51 | 55 | 0.00 | 0.00 | 0 | AJ003057 | 93 - 100 (98) | - |
|  | *Corynebacterium jeikeium* | 0.03 | 0.35 | 21 | 0.00 | 0.66 | 16 | X84250 | 97 - 100 (98) | 0.3 |
|  | *Corynebacterium variabile* | 0.52 | 0.00 | 26 | 0.00 | 0.00 | 0 | AJ222815 | 97 - 98 (97) | - |
|  | *Mycobacterium fallax* | 0.15 | 0.22 | 26 | 0.00 | 0.00 | 0 | AF480600 | 92 - 93 (92) | - |
|  | *Micrococcus luteus* | 0.52 | 0.00 | 18 | 0.20 | 0.00 | 3 | AJ536198 | 96 - 100 (98) | 1.6 |
|  | *Corynebacterium coyleae* | 0.00 | 0.00 | 0 | 1.56 | 0.00 | 19 | X96497 | 95 - 97 (95) | - |
|  | *Segniliparus rugosus* | 0.25 | 0.02 | 17 | 0.00 | 0.00 | 0 | AY608920 | 92 - 94 (93) | - |
|  | *Corynebacterium glaucum* | 0.00 | 0.00 | 0 | 1.25 | 0.00 | 16 | AJ431634 | 93 - 95 (93) | - |
|  | *Bifidobacterium bifidum* | 0.00 | 0.16 | 15 | 0.00 | 0.00 | 0 | U25952 | 97 - 99 (98) | - |
|  | *Corynebacterium kroppenstedtii* | 0.27 | 0.00 | 11 | 0.22 | 0.00 | 3 | Y10077 | 96 - 100 (98) | 1.0 |
|  | *Propionibacterium avidum* | 0.17 | 0.08 | 14 | 0.00 | 0.00 | 0 | AJ003055 | 96 - 100 (98) | - |
|  | *Rothia mucilaginosa* | 0.12 | 0.04 | 8 | 0.14 | 0.08 | 4 | X87758 | 97 - 99 (98) | 0.5 |
|  | *Corynebacterium mucifaciens* | 0.00 | 0.01 | 1 | 0.70 | 0.04 | 10 | Y11200 | 94 - 99 (96) | 0.0 |
|  | *Corynebacterium xerosis* | 0.02 | 0.05 | 4 | 0.00 | 0.29 | 7 | X81914 | 95 - 99 (98) | 0.1 |
|  | *Corynebacterium simulans* | 0.03 | 0.12 | 6 | 0.07 | 0.00 | 1 | AJ012837 | 98 - 100 (99) | 1.6 |
|  | *Gordonia bronchialis* | 0.00 | 0.00 | 0 | 0.21 | 0.17 | 7 | X79287 | 92 - 93 (92) | - |
|  | *Tsukamurella pseudospumae* | 0.00 | 0.00 | 0 | 0.34 | 0.04 | 6 | AY238513 | 93 - 94 (93) | - |
|  | *Actinomyces odontolyticus* | 0.08 | 0.00 | 4 | 0.00 | 0.04 | 1 | AJ234040 | 97 - 100 (98) | 1.0 |
|  | *Dermabacter hominis* | 0.06 | 0.06 | 5 | 0.00 | 0.00 | 0 | X91034 | 94 - 97 (96) | - |
|  | *Actinomyces viscosus* | 0.08 | 0.00 | 3 | 0.00 | 0.04 | 1 | X82453 | 95 - 97 (96) | 0.8 |
|  | *Corynebacterium afermentans* | 0.09 | 0.00 | 3 | 0.00 | 0.00 | 0 | X82054 | 99 - 100 (99) | - |
|  | *Corynebacterium amycolatum* | 0.04 | 0.00 | 2 | 0.07 | 0.00 | 1 | X82057 | 98 - 99 (98) | 0.5 |
|  | *Corynebacterium aurimucosum* | 0.00 | 0.00 | 0 | 0.20 | 0.00 | 3 | AJ309207 | 98 | - |
|  | *Corynebacterium glucuronolyticum* | 0.00 | 0.00 | 0 | 0.07 | 0.04 | 3 | X86688 | 95 - 100 (97) | - |
|  | *Corynebacterium minutissimum* | 0.09 | 0.00 | 3 | 0.00 | 0.00 | 0 | X84678 | 99 | - |
|  | *Corynebacterium singulare* | 0.00 | 0.02 | 2 | 0.07 | 0.00 | 1 | Y10999 | 98 - 99 (98) | 0.5 |
|  | *Arthrobacter nitroguajacolicus* | 0.00 | 0.10 | 2 | 0.00 | 0.00 | 0 | AJ512504 | 99 - 100 (99) | - |
|  | *Corynebacterium halotolerans* | 0.00 | 0.00 | 0 | 0.16 | 0.00 | 2 | AY226509 | 94 | - |
|  | *Corynebacterium striatum* | 0.00 | 0.00 | 0 | 0.00 | 0.08 | 2 | X84442 | 98 - 99 (98) | - |
|  | *Corynebacterium urealyticum* | 0.00 | 0.02 | 2 | 0.00 | 0.00 | 0 | X81913 | 99 | - |
|  | *Mycobacterium porcinum* | 0.02 | 0.01 | 2 | 0.00 | 0.00 | 0 | AY457077 | 92 - 93 (92) | - |
|  | *Actinomyces dentalis* | 0.03 | 0.00 | 1 | 0.00 | 0.00 | 0 | AJ697609 | 98 | - |
|  | *Actinomyces georgiae* | 0.00 | 0.00 | 0 | 0.07 | 0.00 | 1 | X80413 | 97 | - |
|  | *Actinomyces oricola* | 0.03 | 0.00 | 1 | 0.00 | 0.00 | 0 | AJ507295 | 89 | - |
|  | *Actinomyces radingae* | 0.00 | 0.00 | 0 | 0.07 | 0.00 | 1 | X78719 | 97 | - |
|  | *Alloscardovia omnicolens* | 0.02 | 0.00 | 1 | 0.00 | 0.00 | 0 | AM419460 | 99 | - |
|  | *Arcanobacterium haemolyticum* | 0.03 | 0.00 | 1 | 0.00 | 0.00 | 0 | AJ234059 | 90 | - |
|  | *Brachybacterium paraconglomeratum* | 0.00 | 0.00 | 0 | 0.00 | 0.04 | 1 | AJ415377 | 97 | - |
|  | *Brevibacterium casei* | 0.00 | 0.00 | 0 | 0.00 | 0.04 | 1 | AJ251418 | 99 | - |
|  | *Corynebacterium afermentans subsp. lipophilum* | 0.00 | 0.00 | 0 | 0.08 | 0.00 | 1 | X82055 | 96 | - |
|  | *Corynebacterium appendicis* | 0.00 | 0.00 | 0 | 0.08 | 0.00 | 1 | AJ314919 | 97 | - |
|  | *Corynebacterium auris* | 0.00 | 0.00 | 0 | 0.08 | 0.00 | 1 | X81873 | 94 | - |
|  | *Corynebacterium confusum* | 0.00 | 0.01 | 1 | 0.00 | 0.00 | 0 | Y15886 | 97 | - |
|  | *Corynebacterium glutamicum* | 0.00 | 0.01 | 1 | 0.00 | 0.00 | 0 | AF314192 | 91 | - |
|  | *Corynebacterium matruchotii* | 0.03 | 0.00 | 1 | 0.00 | 0.00 | 0 | X82065 | 97 | - |
|  | *Corynebacterium ureicelerivorans* | 0.00 | 0.00 | 0 | 0.00 | 0.05 | 1 | AM397636 | 93 | - |
|  | *Dietzia maris* | 0.00 | 0.00 | 0 | 0.07 | 0.00 | 1 | X79290 | 94 | - |
|  | *Dietzia papillomatosis* | 0.00 | 0.01 | 1 | 0.00 | 0.00 | 0 | AY643401 | 93 | - |
|  | *Friedmanniella antarctica* | 0.03 | 0.00 | 1 | 0.00 | 0.00 | 0 | Z78206 | 94 | - |
|  | *Micropruina glycogenica* | 0.00 | 0.03 | 1 | 0.00 | 0.00 | 0 | AB012607 | 93 | - |
|  | *Propionibacterium propionicum* | 0.00 | 0.01 | 1 | 0.00 | 0.00 | 0 | AJ003058 | 98 | - |
|  | *Pseudonocardia sulfidoxydans* | 0.03 | 0.00 | 1 | 0.00 | 0.00 | 0 | Y08537 | 91 | - |
|  | *Rhodococcus corynebacterioides* | 0.00 | 0.01 | 1 | 0.00 | 0.00 | 0 | AF430066 | 99 | - |
|  | *Streptomyces floridae* | 0.00 | 0.01 | 1 | 0.00 | 0.00 | 0 | AB184656 | 98 | - |
|  | *Streptomyces lavendulae subsp. lavendulae* | 0.00 | 0.00 | 0 | 0.00 | 0.04 | 1 | AB184080 | 96 | - |
|  | *Streptomyces praecox* | 0.00 | 0.01 | 1 | 0.00 | 0.00 | 0 | AB184293 | 99 | - |
|  | *Turicella otitidis* | 0.03 | 0.00 | 1 | 0.00 | 0.00 | 0 | X73976 | 99 | - |
| **Bacteroidetes** | |  |  |  |  |  |  |  |  |  |
|  | *Prevotella melaninogenica* | 0.32 | 0.13 | 18 | 0.22 | 0.04 | 4 | AY323525 | 95 - 100 (98) | 1.2 |
|  | *Prevotella buccalis* | 0.09 | 0.13 | 13 | 0.00 | 0.22 | 5 | L16476 | 97 - 99 (98) | 0.7 |
|  | *Prevotella timonensis* | 0.17 | 0.00 | 11 | 0.07 | 0.00 | 1 | DQ518919 | 98 - 99 (98) | 2.9 |
|  | *Porphyromonas somerae* | 0.02 | 0.06 | 5 | 0.00 | 0.04 | 1 | AY968205 | 90 - 92 (90) | 1.3 |
|  | *Bacteroides stercoris* | 0.00 | 0.05 | 4 | 0.00 | 0.00 | 0 | X83953 | 98 - 99 (98) | - |
|  | *Bacteroides fragilis* | 0.05 | 0.00 | 3 | 0.00 | 0.00 | 0 | CR626927 | 99 - 100 (99) | - |
|  | *Bacteroides plebeius* | 0.00 | 0.05 | 3 | 0.00 | 0.00 | 0 | AB200217 | 91 - 92 (91) | - |
|  | *Capnocytophaga leadbetteri* | 0.04 | 0.01 | 3 | 0.00 | 0.00 | 0 | DQ009623 | 97 - 99 (98) | - |
|  | *Chryseobacterium aquaticum* | 0.05 | 0.00 | 3 | 0.00 | 0.00 | 0 | AM748690 | 96 | - |
|  | *Bacteroides vulgatus* | 0.04 | 0.00 | 2 | 0.00 | 0.00 | 0 | AJ867050 | 99 | - |
|  | *Prevotella bryantii* | 0.07 | 0.00 | 2 | 0.00 | 0.00 | 0 | AJ006457 | 93 - 94 (93) | - |
|  | *Prevotella oulorum* | 0.04 | 0.00 | 2 | 0.00 | 0.00 | 0 | L16472 | 94 - 99 (96) | - |
|  | *Prevotella pallens* | 0.07 | 0.00 | 2 | 0.00 | 0.00 | 0 | Y13105 | 96 - 97 (96) | - |
|  | *Bacteroides caccae* | 0.03 | 0.00 | 1 | 0.00 | 0.00 | 0 | X83951 | 97 | - |
|  | *Bacteroides galacturonicus* | 0.00 | 0.00 | 0 | 0.07 | 0.00 | 1 | DQ497994 | 93 | - |
|  | *Bacteroides intestinalis* | 0.00 | 0.00 | 0 | 0.07 | 0.00 | 1 | AB214328 | 90 | - |
|  | *Capnocytophaga ochracea* | 0.03 | 0.00 | 1 | 0.00 | 0.00 | 0 | U41350 | 94 | - |
|  | *Capnocytophaga sputigena* | 0.00 | 0.00 | 0 | 0.07 | 0.00 | 1 | X67609 | 99 | - |
|  | *Chryseobacterium flavum* | 0.00 | 0.03 | 1 | 0.00 | 0.00 | 0 | EF154516 | 96 | - |
|  | *Hymenobacter norwichensis* | 0.00 | 0.01 | 1 | 0.00 | 0.00 | 0 | AJ549285 | 98 | - |
|  | *Pedobacter aquatilis* | 0.00 | 0.00 | 0 | 0.00 | 0.04 | 1 | AM114396 | 98 | - |
|  | *Pedobacter terricola* | 0.02 | 0.00 | 1 | 0.00 | 0.00 | 0 | EF446147 | 91 | - |
|  | *Porphyromonas endodontalis* | 0.00 | 0.00 | 0 | 0.07 | 0.00 | 1 | AY253728 | 91 | - |
|  | *Porphyromonas gingivalis* | 0.00 | 0.00 | 0 | 0.07 | 0.00 | 1 | AB035459 | 99 | - |
|  | *Prevotella veroralis* | 0.00 | 0.00 | 0 | 0.00 | 0.04 | 1 | L16473 | 96 | - |
| **Cyanobacterium** | |  |  |  |  |  |  |  |  |  |
|  | *Crinalium epipsammum* | 0.03 | 0.02 | 3 | 0.00 | 0.00 | 0 | AB115964 | 88 - 90 (89) | - |
| **Deinococcus** | |  |  |  |  |  |  |  |  |  |
|  | *Thermus thermophilus* | 0.02 | 0.00 | 1 | 0.00 | 0.00 | 0 | X07998 | 100 | - |
| **Firmicutes** | |  |  |  |  |  |  |  |  |  |
|  | *Staphylococcus aureus subsp. aureus* | 0.10 | 46.08 | 4966 | 0.07 | 39.24 | 922 | L36472 | 96 - 100 (99) | 1.4 |
|  | *Staphylococcus epidermidis* | 43.74 | 8.21 | 3517 | 41.57 | 7.91 | 796 | D83363 | 94 - 100 (99) | 1.2 |
|  | *Staphylococcus simulans* | 0.36 | 2.56 | 282 | 0.79 | 1.97 | 53 | D83373 | 94 - 100 (98) | 1.4 |
|  | *Peptoniphilus harei* | 1.77 | 1.32 | 203 | 4.06 | 2.08 | 109 | Y07839 | 90 - 100 (94) | 0.5 |
|  | *Anaerococcus octavius* | 1.10 | 1.05 | 152 | 3.75 | 3.19 | 128 | Y07841 | 90 - 100 (95) | 0.3 |
|  | *Streptococcus pyogenes* | 0.00 | 1.26 | 150 | 0.00 | 2.43 | 52 | AB002521 | 96 - 100 (98) | 0.8 |
|  | *Finegoldia magna* | 1.08 | 0.71 | 117 | 2.53 | 1.13 | 64 | AF542227 | 97 - 100 (99) | 0.5 |
|  | *Staphylococcus caprae* | 0.11 | 1.11 | 140 | 0.07 | 0.73 | 18 | AB009935 | 98 - 100 (99) | 2.0 |
|  | *Dolosigranulum pigrum* | 0.20 | 0.77 | 53 | 0.13 | 1.69 | 42 | X70907 | 94 - 99 (97) | 0.3 |
|  | *Staphylococcus saprophyticus subsp. saprophyticus* | 0.03 | 0.83 | 73 | 0.00 | 0.48 | 8 | AP008934 | 94 - 100 (98) | 2.4 |
|  | *Streptococcus mitis* | 0.38 | 0.12 | 28 | 2.04 | 0.46 | 40 | AF003929 | 95 - 100 (99) | 0.2 |
|  | *Staphylococcus haemolyticus* | 0.75 | 0.16 | 52 | 0.60 | 0.14 | 11 | X66100 | 97 - 100 (98) | 1.2 |
|  | *Staphylococcus warneri* | 0.99 | 0.00 | 56 | 0.33 | 0.00 | 6 | L37603 | 98 - 100 (99) | 2.4 |
|  | *Staphylococcus lugdunensis* | 1.10 | 0.08 | 33 | 1.29 | 0.04 | 20 | AB009941 | 97 - 100 (99) | 0.4 |
|  | *Pediococcus acidilactici* | 0.63 | 0.00 | 31 | 1.12 | 0.00 | 15 | AJ305320 | 96 - 100 (98) | 0.5 |
|  | *Anaerococcus vaginalis* | 0.16 | 0.16 | 23 | 0.64 | 0.45 | 20 | AF542229 | 90 - 99 (93) | 0.3 |
|  | *Enterococcus faecalis* | 0.00 | 0.30 | 29 | 0.07 | 0.56 | 13 | AB012212 | 96 - 100 (99) | 0.6 |
|  | *Staphylococcus hominis subsp. novobiosepticus* | 0.06 | 0.48 | 25 | 0.07 | 0.65 | 16 | AB233326 | 98 - 100 (99) | 0.4 |
|  | *Bacillus thuringiensis* | 0.00 | 0.36 | 40 | 0.00 | 0.00 | 0 | D16281 | 98 - 100 (99) | - |
|  | *Streptococcus parasanguinis* | 0.34 | 0.14 | 18 | 0.70 | 0.29 | 17 | AF003933 | 95 - 99 (97) | 0.3 |
|  | *Streptococcus salivarius* | 0.24 | 0.31 | 24 | 0.28 | 0.25 | 10 | AY188352 | 98 - 100 (99) | 0.6 |
|  | *Staphylococcus simiae* | 0.00 | 0.06 | 7 | 0.07 | 0.80 | 16 | AY727530 | 92 - 100 (97) | 0.1 |
|  | *Bacillus cereus* | 0.00 | 0.02 | 2 | 0.07 | 0.74 | 19 | AE016877 | 96 - 99 (98) | 0.0 |
|  | *Streptococcus sanguinis* | 0.03 | 0.16 | 8 | 0.07 | 0.42 | 11 | AF003928 | 95 - 100 (98) | 0.2 |
|  | *Granulicatella adiacens* | 0.16 | 0.06 | 11 | 0.14 | 0.13 | 5 | D50540 | 95 - 99 (96) | 0.6 |
|  | *Staphylococcus sciuri subsp. sciuri* | 0.00 | 0.17 | 14 | 0.00 | 0.11 | 2 | AJ421446 | 98 - 100 (99) | 1.8 |
|  | *Streptococcus infantis* | 0.16 | 0.00 | 6 | 0.60 | 0.08 | 10 | AY485603 | 97 - 99 (98) | 0.2 |
|  | *Streptococcus oralis* | 0.19 | 0.15 | 13 | 0.07 | 0.08 | 3 | AY485602 | 98 - 99 (98) | 1.1 |
|  | *Staphylococcus xylosus* | 0.00 | 0.01 | 1 | 0.00 | 0.59 | 14 | D83374 | 93 - 99 (98) | 0.0 |
|  | *Streptococcus vestibularis* | 0.24 | 0.01 | 13 | 0.00 | 0.00 | 0 | AY188353 | 98 - 99 (98) | - |
|  | *Dialister propionicifaciens* | 0.03 | 0.05 | 5 | 0.14 | 0.09 | 4 | AY850119 | 99 - 100 (99) | 0.3 |
|  | *Lactobacillus plantarum subsp. plantarum* | 0.14 | 0.00 | 7 | 0.15 | 0.00 | 2 | AJ965482 | 97 - 100 (98) | 0.9 |
|  | *Staphylococcus hominis subsp. hominis* | 0.05 | 0.04 | 6 | 0.07 | 0.09 | 3 | X66101 | 97 - 100 (98) | 0.5 |
|  | *Staphylococcus auricularis* | 0.00 | 0.06 | 5 | 0.00 | 0.12 | 3 | D83358 | 93 - 100 (97) | 0.4 |
|  | *Abiotrophia defectiva* | 0.03 | 0.03 | 2 | 0.22 | 0.04 | 4 | D50541 | 95 - 99 (97) | 0.1 |
|  | *Megasphaera micronuciformis* | 0.04 | 0.05 | 6 | 0.00 | 0.00 | 0 | AF473834 | 91 - 92 (91) | - |
|  | *Streptococcus peroris* | 0.09 | 0.07 | 6 | 0.00 | 0.00 | 0 | AB008314 | 96 - 98 (96) | - |
|  | *Streptococcus pseudopneumoniae* | 0.01 | 0.00 | 1 | 0.27 | 0.00 | 5 | AY612844 | 98 - 100 (99) | 0.1 |
|  | *Streptococcus australis* | 0.06 | 0.00 | 3 | 0.07 | 0.04 | 2 | AY485604 | 96 - 99 (97) | 0.4 |
|  | *Veillonella atypica* | 0.14 | 0.00 | 5 | 0.00 | 0.00 | 0 | AF439641 | 98 - 100 (99) | - |
|  | *Veillonella dispar* | 0.13 | 0.00 | 5 | 0.00 | 0.00 | 0 | AF439639 | 97 - 99 (98) | - |
|  | *Veillonella parvula* | 0.03 | 0.10 | 5 | 0.00 | 0.00 | 0 | AY995767 | 95 - 99 (97) | - |
|  | *Enterococcus durans* | 0.08 | 0.00 | 4 | 0.00 | 0.00 | 0 | AJ276354 | 99 | - |
|  | *Streptococcus gordonii* | 0.05 | 0.00 | 2 | 0.07 | 0.04 | 2 | AF003931 | 99 - 100 (99) | 0.3 |
|  | *Dialister micraerophilus* | 0.00 | 0.00 | 0 | 0.07 | 0.09 | 3 | AF473837 | 90 - 91 (90) | - |
|  | *Peptoniphilus ivorii* | 0.06 | 0.00 | 2 | 0.07 | 0.00 | 1 | Y07840 | 95 - 97 (95) | 0.5 |
|  | *Atopostipes suicloacalis* | 0.06 | 0.00 | 2 | 0.00 | 0.00 | 0 | AF445248 | 94 | - |
|  | *Clostridium ultunense* | 0.03 | 0.00 | 2 | 0.00 | 0.00 | 0 | Z69293 | 85 | - |
|  | *Gemella sanguinis* | 0.02 | 0.00 | 1 | 0.07 | 0.00 | 1 | Y13364 | 99 - 100 (99) | 0.3 |
|  | *Ignavigranum ruoffiae* | 0.06 | 0.00 | 2 | 0.00 | 0.00 | 0 | Y16426 | 88 - 89 (88) | - |
|  | *Lactobacillus rossiae* | 0.04 | 0.00 | 2 | 0.00 | 0.00 | 0 | AJ564009 | 94 | - |
|  | *Moryella indoligenes* | 0.00 | 0.00 | 0 | 0.00 | 0.08 | 2 | DQ377947 | 94 - 95 (94) | - |
|  | *Ruminococcus torques* | 0.04 | 0.00 | 2 | 0.00 | 0.00 | 0 | L76604 | 93 | - |
|  | *Selenomonas sputigena* | 0.03 | 0.03 | 2 | 0.00 | 0.00 | 0 | AF287793 | 96 - 98 (97) | - |
|  | *Soehngenia saccharolytica* | 0.04 | 0.00 | 2 | 0.00 | 0.00 | 0 | AY353956 | 95 | - |
|  | *Staphylococcus capitis subsp. urealyticus* | 0.00 | 0.00 | 0 | 0.00 | 0.09 | 2 | AB009937 | 98 - 99 (98) | - |
|  | *Staphylococcus pasteuri* | 0.00 | 0.02 | 2 | 0.00 | 0.00 | 0 | AB009944 | 96 - 97 (96) | - |
|  | *Streptococcus anginosus* | 0.06 | 0.00 | 2 | 0.00 | 0.00 | 0 | AF104678 | 98 - 99 (98) | - |
|  | *Streptococcus cristatus* | 0.00 | 0.00 | 0 | 0.14 | 0.00 | 2 | AY188347 | 97 - 98 (97) | - |
|  | *Anaerococcus hydrogenalis* | 0.03 | 0.00 | 1 | 0.00 | 0.00 | 0 | D14140 | 99 | - |
|  | *Anaerococcus lactolyticus* | 0.02 | 0.00 | 1 | 0.00 | 0.00 | 0 | AF542233 | 94 | - |
|  | *Anaerococcus prevotii* | 0.00 | 0.02 | 1 | 0.00 | 0.00 | 0 | AF542232 | 98 | - |
|  | *Anaerococcus tetradius* | 0.00 | 0.00 | 0 | 0.08 | 0.00 | 1 | AF542234 | 93 | - |
|  | *Bacillus aerius* | 0.00 | 0.01 | 1 | 0.00 | 0.00 | 0 | AJ831843 | 98 | - |
|  | *Bacillus sonorensis* | 0.00 | 0.03 | 1 | 0.00 | 0.00 | 0 | AF302118 | 99 | - |
|  | *Bacillus weihenstephanensis* | 0.00 | 0.00 | 0 | 0.00 | 0.04 | 1 | AB021199 | 97 | - |
|  | *Caldicellulosiruptor acetigenus* | 0.00 | 0.01 | 1 | 0.00 | 0.00 | 0 | AY772476 | 98 | - |
|  | *Catonella morbi* | 0.00 | 0.03 | 1 | 0.00 | 0.00 | 0 | X87151 | 99 | - |
|  | *Clostridium bolteae* | 0.00 | 0.00 | 0 | 0.07 | 0.00 | 1 | AJ508452 | 93 | - |
|  | *Clostridium lactatifermentans* | 0.00 | 0.01 | 1 | 0.00 | 0.00 | 0 | AY033434 | 91 | - |
|  | *Clostridium leptum* | 0.00 | 0.01 | 1 | 0.00 | 0.00 | 0 | AJ305238 | 89 | - |
|  | *Desulfitibacter alkalitolerans* | 0.00 | 0.01 | 1 | 0.00 | 0.00 | 0 | AY538171 | 96 | - |
|  | *Enterococcus pallens* | 0.00 | 0.00 | 0 | 0.00 | 0.05 | 1 | DQ411812 | 93 | - |
|  | *Eubacterium angustum* | 0.00 | 0.01 | 1 | 0.00 | 0.00 | 0 | L34612 | 85 | - |
|  | *Eubacterium rectale* | 0.02 | 0.00 | 1 | 0.00 | 0.00 | 0 | L34627 | 96 | - |
|  | *Eubacterium siraeum* | 0.00 | 0.00 | 0 | 0.07 | 0.00 | 1 | L34625 | 97 | - |
|  | *Gemella haemolysans* | 0.00 | 0.02 | 1 | 0.00 | 0.00 | 0 | L14326 | 99 | - |
|  | *Gemella morbillorum* | 0.00 | 0.00 | 0 | 0.07 | 0.00 | 1 | L14327 | 99 | - |
|  | *Geobacillus tepidamans* | 0.00 | 0.02 | 1 | 0.00 | 0.00 | 0 | AY563003 | 98 | - |
|  | *Howardella ureilytica* | 0.02 | 0.00 | 1 | 0.00 | 0.00 | 0 | DQ925472 | 94 | - |
|  | *Lactobacillus panis* | 0.00 | 0.00 | 0 | 0.07 | 0.00 | 1 | X94230 | 95 | - |
|  | *Lactobacillus rhamnosus* | 0.00 | 0.02 | 1 | 0.00 | 0.00 | 0 | D16552 | 100 | - |
|  | *Leuconostoc mesenteroides subsp. mesenteroides* | 0.03 | 0.00 | 1 | 0.00 | 0.00 | 0 | CP000414 | 99 | - |
|  | *Oceanobacillus iheyensis* | 0.00 | 0.01 | 1 | 0.00 | 0.00 | 0 | AB010863 | 87 | - |
|  | *Oribacterium sinus* | 0.00 | 0.00 | 0 | 0.07 | 0.00 | 1 | AY323228 | 95 | - |
|  | *Roseburia inulinivorans* | 0.00 | 0.01 | 1 | 0.00 | 0.00 | 0 | AJ270473 | 100 | - |
|  | *Ruminococcus schinkii* | 0.02 | 0.00 | 1 | 0.00 | 0.00 | 0 | X94965 | 96 | - |
|  | *Selenomonas infelix* | 0.03 | 0.00 | 1 | 0.00 | 0.00 | 0 | AF287802 | 92 | - |
|  | *Sporanaerobacter acetigenes* | 0.00 | 0.00 | 0 | 0.07 | 0.00 | 1 | AF358114 | 89 | - |
|  | *Staphylococcus cohnii subsp. urealyticus* | 0.00 | 0.00 | 0 | 0.00 | 0.05 | 1 | AB009936 | 96 | - |
|  | *Staphylococcus hyicus* | 0.00 | 0.00 | 0 | 0.00 | 0.05 | 1 | D83368 | 98 | - |
|  | *Staphylococcus saccharolyticus* | 0.00 | 0.01 | 1 | 0.00 | 0.00 | 0 | L37602 | 100 | - |
|  | *Streptococcus pneumoniae* | 0.00 | 0.01 | 1 | 0.00 | 0.00 | 0 | AF003930 | 99 | - |
|  | *Streptococcus sinensis* | 0.03 | 0.00 | 1 | 0.00 | 0.00 | 0 | AF432856 | 99 | - |
|  | *Veillonella denticariosi* | 0.03 | 0.00 | 1 | 0.00 | 0.00 | 0 | EF185167 | 98 | - |
| **Fusobacteria** | |  |  |  |  |  |  |  |  |  |
|  | *Fusobacterium periodonticum* | 0.07 | 0.01 | 3 | 0.00 | 0.00 | 0 | X55405 | 95 - 97 (96) | - |
|  | *Fusobacterium canifelinum* | 0.05 | 0.00 | 2 | 0.00 | 0.00 | 0 | AY162221 | 98 | - |
|  | *Fusobacterium nucleatum subsp. polymorphum* | 0.00 | 0.00 | 0 | 0.15 | 0.00 | 2 | AF287812 | 97 - 98 (97) | - |
|  | *Fusobacterium nucleatum subsp. nucleatum* | 0.00 | 0.00 | 0 | 0.00 | 0.04 | 1 | AE009951 | 97 | - |
|  | *Leptotrichia wadei* | 0.00 | 0.03 | 1 | 0.00 | 0.00 | 0 | AY029802 | 96 | - |
| **Proteobacteria** | |  |  |  |  |  |  |  |  |  |
|  | *Enterobacter ludwigii* | 0.14 | 5.35 | 349 | 0.07 | 4.07 | 100 | AJ853891 | 98 - 100 (99) | 0.9 |
|  | *Pseudomonas xanthomarina* | 0.02 | 3.11 | 349 | 0.00 | 0.00 | 0 | AB176954 | 94 - 100 (99) | - |
|  | *Shigella sonnei* | 1.71 | 3.50 | 153 | 0.45 | 0.00 | 6 | X80726 | 94 - 100 (99) | 6.7 |
|  | *Pseudomonas luteola* | 0.00 | 0.67 | 56 | 0.00 | 1.07 | 21 | D84002 | 94 - 100 (98) | 0.7 |
|  | *Simonsiella muelleri* | 0.00 | 0.00 | 0 | 3.83 | 0.50 | 67 | AF328147 | 89 - 92 (90) | - |
|  | *Enterobacter cowanii* | 0.00 | 0.00 | 0 | 0.00 | 3.47 | 64 | AJ508303 | 95 - 98 (97) | - |
|  | *Pseudomonas putida* | 0.00 | 0.00 | 0 | 0.13 | 2.15 | 54 | D84020 | 98 - 99 (98) | - |
|  | *Acinetobacter lwoffii* | 0.87 | 0.00 | 30 | 1.36 | 0.00 | 20 | X81665 | 98 - 100 (99) | 0.4 |
|  | *Serratia marcescens subsp. sakuensis* | 0.00 | 0.00 | 0 | 0.00 | 1.82 | 42 | AB061685 | 98 - 99 (98) | - |
|  | *Xanthomonas vesicatoria* | 0.00 | 0.90 | 35 | 0.00 | 0.00 | 0 | Y10761 | 98 - 100 (99) | - |
|  | *Serratia marcescens subsp. marcescens* | 0.02 | 2.08 | 9 | 0.00 | 0.91 | 21 | AJ233431 | 93 - 100 (98) | 0.1 |
|  | *Shigella flexneri* | 0.00 | 0.00 | 0 | 2.08 | 0.05 | 29 | X96963 | 96 - 99 (98) | - |
|  | *Microvirgula aerodenitrificans* | 2.65 | 0.00 | 24 | 0.00 | 0.00 | 0 | U89333 | 87 - 94 (88) | - |
|  | *Pseudomonas asplenii* | 0.00 | 0.00 | 0 | 0.47 | 0.62 | 22 | AB021397 | 94 - 99 (98) | - |
|  | *Haemophilus ducreyi* | 0.43 | 0.25 | 20 | 0.00 | 0.00 | 0 | M63900 | 98 - 99 (98) | - |
|  | *Herbaspirillum huttiense* | 0.00 | 0.00 | 0 | 1.35 | 0.00 | 20 | AB021366 | 88 - 89 (88) | - |
|  | *Haemophilus paraphrohaemolyticus* | 0.00 | 0.00 | 0 | 0.54 | 0.47 | 19 | M75076 | 95 - 96 (95) | - |
|  | *Paracoccus yeei* | 0.35 | 0.00 | 12 | 0.48 | 0.00 | 7 | AY014173 | 99 - 100 (99) | 0.4 |
|  | *Bacteroides ureolyticus* | 0.00 | 0.10 | 8 | 0.22 | 0.22 | 8 | L04321 | 96 - 99 (97) | 0.3 |
|  | *Pseudomonas stutzeri* | 0.02 | 0.12 | 12 | 0.00 | 0.13 | 3 | AF094748 | 97 - 100 (98) | 1.0 |
|  | *Aquabacterium citratiphilum* | 0.22 | 0.00 | 13 | 0.00 | 0.00 | 0 | AF035050 | 96 - 98 (96) | - |
|  | *Rhodocyclus tenuis* | 0.20 | 0.05 | 12 | 0.00 | 0.00 | 0 | D16208 | 85 - 86 (85) | - |
|  | *Propionivibrio dicarboxylicus* | 0.18 | 0.04 | 11 | 0.00 | 0.00 | 0 | Y17601 | 94 - 98 (97) | - |
|  | *Pannonibacter phragmitetus* | 0.17 | 0.04 | 10 | 0.00 | 0.00 | 0 | AJ400704 | 94 - 97 (95) | - |
|  | *Acinetobacter schindleri* | 0.13 | 0.00 | 8 | 0.07 | 0.00 | 1 | AJ278311 | 98 - 99 (98) | 2.1 |
|  | *Pasteurella mairii* | 0.00 | 0.09 | 9 | 0.00 | 0.00 | 0 | AY362923 | 96 | - |
|  | *Salmonella enterica subsp. arizonae* | 0.18 | 0.00 | 9 | 0.00 | 0.00 | 0 | AF008580 | 98 | - |
|  | *Citrobacter farmeri* | 0.16 | 0.00 | 8 | 0.00 | 0.00 | 0 | AF025371 | 97 - 98 (97) | - |
|  | *Haemophilus parainfluenzae* | 0.11 | 0.01 | 5 | 0.07 | 0.08 | 3 | AY362908 | 97 - 99 (97) | 0.4 |
|  | *Neisseria flavescens* | 0.15 | 0.03 | 6 | 0.00 | 0.04 | 1 | L06168 | 97 - 98 (97) | 1.6 |
|  | *Aquabacterium parvum* | 0.00 | 0.00 | 0 | 0.07 | 0.21 | 6 | AF035052 | 98 | - |
|  | *Enterobacter kobei* | 0.00 | 0.02 | 2 | 0.00 | 0.17 | 4 | AJ508301 | 98 - 100 (98) | 0.1 |
|  | *Herbaspirillum putei* | 0.00 | 0.00 | 0 | 0.41 | 0.00 | 6 | AB109890 | 86 | - |
|  | *Pseudomonas mosselii* | 0.10 | 0.00 | 6 | 0.00 | 0.00 | 0 | AF072688 | 99 | - |
|  | *Serratia ureilytica* | 0.00 | 0.00 | 0 | 0.00 | 0.26 | 6 | AJ854062 | 98 - 99 (98) | - |
|  | *Shigella dysenteriae* | 0.00 | 0.00 | 0 | 0.45 | 0.00 | 6 | X96966 | 99 | - |
|  | *Acinetobacter junii* | 0.00 | 0.10 | 4 | 0.00 | 0.04 | 1 | X81664 | 99 - 100 (99) | 1.0 |
|  | *Moraxella (subgen. Moraxella Lwoff 1939) lacunata* | 0.00 | 0.03 | 2 | 0.00 | 0.13 | 3 | D64049 | 97 - 98 (97) | 0.2 |
|  | *Neisseria animaloris* | 0.03 | 0.00 | 1 | 0.20 | 0.00 | 3 | DQ006842 | 88 - 92 (89) | 0.1 |
|  | *Rhizobium radiobacter* | 0.00 | 0.08 | 3 | 0.00 | 0.04 | 1 | AB247615 | 98 - 100 (99) | 0.8 |
|  | *Xanthomonas theicola* | 0.00 | 0.00 | 0 | 0.00 | 0.17 | 4 | Y10763 | 99 | - |
|  | *Delftia tsuruhatensis* | 0.00 | 0.00 | 0 | 0.21 | 0.00 | 3 | AB075017 | 99 - 100 (99) | - |
|  | *Haemophilus pittmaniae* | 0.00 | 0.00 | 0 | 0.07 | 0.09 | 3 | AJ290755 | 94 | - |
|  | *Janthinobacterium lividum* | 0.00 | 0.05 | 2 | 0.00 | 0.04 | 1 | Y08846 | 98 - 99 (98) | 0.5 |
|  | *Neisseria lactamica* | 0.03 | 0.00 | 1 | 0.00 | 0.08 | 2 | AJ239286 | 93 - 96 (95) | 0.1 |
|  | *Pseudomonas pseudoalcaligenes* | 0.00 | 0.00 | 0 | 0.20 | 0.00 | 3 | Z76666 | 97 | - |
|  | *Roseateles aquatilis* | 0.00 | 0.00 | 0 | 0.14 | 0.04 | 3 | AM501446 | 99 - 100 (99) | - |
|  | *Sphingomonas faeni* | 0.00 | 0.08 | 3 | 0.00 | 0.00 | 0 | AJ429239 | 98 - 100 (99) | - |
|  | *Aurantimonas altamirensis* | 0.00 | 0.00 | 0 | 0.14 | 0.00 | 2 | DQ372921 | 99 | - |
|  | *Enterobacter aerogenes* | 0.00 | 0.00 | 0 | 0.00 | 0.08 | 2 | AB004750 | 99 | - |
|  | *Enterobacter asburiae* | 0.00 | 0.00 | 0 | 0.00 | 0.11 | 2 | AB004744 | 97 - 99 (98) | - |
|  | *Erwinia persicina* | 0.00 | 0.02 | 2 | 0.00 | 0.00 | 0 | U80205 | 98 | - |
|  | *Naxibacter alkalitolerans* | 0.03 | 0.00 | 2 | 0.00 | 0.00 | 0 | AY679161 | 99 | - |
|  | *Paracoccus marcusii* | 0.00 | 0.00 | 0 | 0.14 | 0.00 | 2 | Y12703 | 99 | - |
|  | *Ralstonia insidiosa* | 0.00 | 0.00 | 0 | 0.07 | 0.04 | 2 | AF488779 | 99 | - |
|  | *Ralstonia mannitolilytica* | 0.00 | 0.04 | 2 | 0.00 | 0.00 | 0 | AJ270258 | 99 | - |
|  | *Uruburuella suis* | 0.05 | 0.00 | 2 | 0.00 | 0.00 | 0 | AJ586614 | 87 | - |
|  | *Yersinia enterocolitica subsp. enterocolitica* | 0.07 | 0.00 | 2 | 0.00 | 0.00 | 0 | AF366378 | 97 | - |
|  | *Acinetobacter calcoaceticus* | 0.00 | 0.03 | 1 | 0.00 | 0.00 | 0 | AJ888983 | 97 | - |
|  | *Acinetobacter ursingii* | 0.00 | 0.00 | 0 | 0.07 | 0.00 | 1 | AJ275038 | 99 | - |
|  | *Actinobacillus indolicus* | 0.02 | 0.00 | 1 | 0.00 | 0.00 | 0 | AY362891 | 93 | - |
|  | *Afipia broomeae* | 0.00 | 0.00 | 0 | 0.07 | 0.00 | 1 | U87759 | 97 | - |
|  | *Aquabacterium commune* | 0.00 | 0.03 | 1 | 0.00 | 0.00 | 0 | AF035054 | 97 | - |
|  | *Blastobacter aggregatus* | 0.02 | 0.00 | 1 | 0.00 | 0.00 | 0 | X73041 | 99 | - |
|  | *Campylobacter concisus* | 0.03 | 0.00 | 1 | 0.00 | 0.00 | 0 | DQ174166 | 95 | - |
|  | *Campylobacter gracilis* | 0.00 | 0.00 | 0 | 0.07 | 0.00 | 1 | DQ174168 | 99 | - |
|  | *Citrobacter murliniae* | 0.03 | 0.00 | 1 | 0.00 | 0.00 | 0 | AF025369 | 99 | - |
|  | *Comamonas testosteroni* | 0.03 | 0.00 | 1 | 0.00 | 0.00 | 0 | M11224 | 100 | - |
|  | *Cronobacter dublinensis subsp. lausannensis* | 0.00 | 0.00 | 0 | 0.00 | 0.04 | 1 | EF059841 | 98 | - |
|  | *Cronobacter muytjensii* | 0.00 | 0.01 | 1 | 0.00 | 0.00 | 0 | EF059845 | 100 | - |
|  | *Cupriavidus basilensis* | 0.03 | 0.00 | 1 | 0.00 | 0.00 | 0 | AF312022 | 99 | - |
|  | *Cupriavidus respiraculi* | 0.00 | 0.03 | 1 | 0.00 | 0.00 | 0 | AF500583 | 99 | - |
|  | *Duganella zoogloeoides* | 0.00 | 0.00 | 0 | 0.00 | 0.04 | 1 | D14256 | 99 | - |
|  | *Erwinia amylovora* | 0.00 | 0.01 | 1 | 0.00 | 0.00 | 0 | AJ233410 | 98 | - |
|  | *Haemophilus aegyptius* | 0.00 | 0.00 | 0 | 0.00 | 0.04 | 1 | AY362905 | 96 | - |
|  | *Halomonas subterranea* | 0.00 | 0.03 | 1 | 0.00 | 0.00 | 0 | EF144148 | 98 | - |
|  | *Kingella kingae* | 0.00 | 0.00 | 0 | 0.07 | 0.00 | 1 | AY551999 | 88 | - |
|  | *Klebsiella oxytoca* | 0.00 | 0.00 | 0 | 0.07 | 0.00 | 1 | AF129440 | 99 | - |
|  | *Klebsiella pneumoniae subsp. rhinoscleromatis* | 0.00 | 0.00 | 0 | 0.00 | 0.05 | 1 | Y17657 | 95 | - |
|  | *Maricaulis virginensis* | 0.02 | 0.00 | 1 | 0.00 | 0.00 | 0 | AJ301667 | 95 | - |
|  | *Methylobacterium iners* | 0.00 | 0.03 | 1 | 0.00 | 0.00 | 0 | EF174497 | 94 | - |
|  | *Methylobacterium tardum* | 0.03 | 0.00 | 1 | 0.00 | 0.00 | 0 | AB252208 | 99 | - |
|  | *Neisseria elongata subsp. elongata* | 0.22 | 0.00 | 1 | 0.00 | 0.00 | 0 | L06171 | 88 | - |
|  | *Neisseria weaveri* | 0.00 | 0.00 | 0 | 0.00 | 0.04 | 1 | L10738 | 95 | - |
|  | *Paludibacterium yongneupense* | 0.02 | 0.00 | 1 | 0.00 | 0.00 | 0 | AM396358 | 86 | - |
|  | *Pelomonas aquatica* | 0.00 | 0.00 | 0 | 0.07 | 0.00 | 1 | AM501435 | 99 | - |
|  | *Pseudomonas agarici* | 0.02 | 0.00 | 1 | 0.00 | 0.00 | 0 | Z76652 | 98 | - |
|  | *Pseudomonas cannabina* | 0.00 | 0.00 | 0 | 0.00 | 0.04 | 1 | AJ492827 | 99 | - |
|  | *Pseudomonas duriflava* | 0.00 | 0.05 | 1 | 0.00 | 0.00 | 0 | EU046271 | 96 | - |
|  | *Pseudomonas kilonensis* | 0.00 | 0.00 | 0 | 0.00 | 0.04 | 1 | AJ292426 | 99 | - |
|  | *Pseudomonas plecoglossicida* | 0.00 | 0.01 | 1 | 0.00 | 0.00 | 0 | AB009457 | 100 | - |
|  | *Rhizobium vitis* | 0.02 | 0.00 | 1 | 0.00 | 0.00 | 0 | U45329 | 97 | - |
|  | *Salmonella bongori* | 0.00 | 0.00 | 0 | 0.00 | 0.05 | 1 | AF029227 | 97 | - |
|  | *Salmonella enterica subsp. salamae* | 0.00 | 0.01 | 1 | 0.00 | 0.00 | 0 | EU014685 | 93 | - |
|  | *Serratia odorifera* | 0.00 | 0.00 | 0 | 0.00 | 0.04 | 1 | AJ233432 | 95 | - |
|  | *Sphingobium amiense* | 0.02 | 0.00 | 1 | 0.00 | 0.00 | 0 | AB047364 | 99 | - |
|  | *Sphingomonas echinoides* | 0.00 | 0.01 | 1 | 0.00 | 0.00 | 0 | AB021370 | 99 | - |
|  | *Sphingomonas mucosissima* | 0.02 | 0.00 | 1 | 0.00 | 0.00 | 0 | AM229669 | 99 | - |
|  | *Sphingomonas terrae* | 0.03 | 0.00 | 1 | 0.00 | 0.00 | 0 | D13727 | 92 | - |
|  | *Sphingomonas ursincola* | 0.00 | 0.00 | 0 | 0.00 | 0.04 | 1 | AB024289 | 99 | - |
|  | *Stenotrophomonas acidaminiphila* | 0.03 | 0.00 | 1 | 0.00 | 0.00 | 0 | AF273080 | 100 | - |
|  | *Xanthomonas cynarae* | 0.00 | 0.00 | 0 | 0.00 | 0.04 | 1 | AF208315 | 99 | - |
|  | *Xanthomonas vasicola* | 0.00 | 0.00 | 0 | 0.00 | 0.04 | 1 | Y10755 | 99 | - |
| **Tenericutes** | |  |  |  |  |  |  |  |  |  |
|  | Ureaplasma parvum | 0.03 | 0.00 | 2 | 0.00 | 0.00 | 0 | AF073456 | 99 - 100 (99) | - |
|  | Total | 100% | 100% | 14322 | 100% | 100% | 3753 |  |  |  |
|  |  |  |  |  |  |  |  |  |  |  |
| ^1^ Sequencing platform used. Pyrosequencing = Roche 454 GS-FLX; Sanger = MegaBACE1000. | | | | | | | | |  |  |
| ^2^ Classification of samples by *Staphylococcus aureus*-specific *fem*A gene PCR. | | | | | | |  |  |  |  |
| ^4^ Identity of microorganisms inferred from highest bit-score in BLAST query. Hits with Blast %IDs < 97 are named only to the genus level. | | | | | | | | | | |
| ^5^ Number of sequences analyzed for grouping. | | |  |  |  |  |  |  |  |  |
| ^6^ GenBank accession number for top Blast hit. | | |  |  |  |  |  |  |  |  |
| ^7^ Mean Blast percent identity score for top Blast hit | | | |  |  |  |  |  |  |  |
| ^8^ Percent of sequences classified as species/genus for *fem*A-based category. Columns sum to 100% | | | | | | | | |  |  |
| ^9^ Lower abundance sequences (< 10% of total) were omitted to simplify the table | | | | | | |  |  |  |  |
